# Renal association clinical practice guideline in post-operative care in the kidney transplant recipient

**DOI:** 10.1186/s12882-017-0553-2

**Published:** 2017-06-02

**Authors:** Richard J. Baker, Patrick B. Mark, Rajan K. Patel, Kate K. Stevens, Nicholas Palmer

**Affiliations:** 1 Renal Unit, St. James’s University Hospital, Leeds, England; 2 Glasgow Renal and Transplant Unit, Queen Elizabeth University Hospital, Glasgow, Scotland; 3 British Kidney Patient Association, Alton, UK

## Abstract

These guidelines cover the care of patients from the period following kidney transplantation until the transplant is no longer working or the patient dies. During the early phase prevention of acute rejection and infection are the priority. After around 3–6 months, the priorities change to preservation of transplant function and avoiding the long-term complications of immunosuppressive medication (the medication used to suppress the immune system to prevent rejection). The topics discussed include organization of outpatient follow up, immunosuppressive medication, treatment of acute and chronic rejection, and prevention of complications. The potential complications discussed include heart disease, infection, cancer, bone disease and blood disorders. There is also a section on contraception and reproductive issues.

Immediately after the introduction there is a statement of all the recommendations. These recommendations are written in a language that we think should be understandable by many patients, relatives, carers and other interested people. Consequently we have not reworded or restated them in this lay summary. They are graded 1 or 2 depending on the strength of the recommendation by the authors, and AD depending on the quality of the evidence that the recommendation is based on.

## Introduction

This document is intended for those engaged in the care of kidney transplant recipients (KTR) who are non-experts. With increasing efforts to deliver health care locally, many renal transplant recipients are followed up in centres remote from the main surgical transplant unit. At the same time, transplantation medicine has evolved into an increasingly complex and specialised field of nephrology. The following guidelines reflect this alteration in clinical practice and are intended for those healthcare professionals who look after renal transplant patients. They are also intended to be useful to both medical and surgical trainees, general practitioners, nurse specialists and other associated healthcare professionals involved in the care of renal transplant patients.

These guidelines cover the period after renal transplantation, specifically from initial hospital discharge until graft failure or patient death. The management of KTR can be divided into two phases:

- an early post-operative phase when prevention of acute rejection, optimization of graft function and prevention of opportunistic infection are paramount
- a later phase when the aims are to preserve good graft function, ensure adherence to medication, and prevent the long-term consequences of immunosuppression – malignancy, infection and premature cardiovascular disease.

The transition between these two phases occurs around 3–6 months after transplantation at the time when the progressive, protocolised reduction in immunosuppression following transplantation reaches long-term maintenance levels. Management of the early and late phase complications of transplantation requires monitoring at reducing frequency, awareness of complications, access to investigation, and strategies for prevention and treatment of complications (ranging from early acute rejection, to late cardiovascular disease). There are regional differences in demographics, risk and organisation of services. The priority is agreement of local strategies for post-transplant management.

These guidelines are designed to complement those previously published relating to pre-transplant care.

It should be noted that other comprehensive guidelines have been published and reference will be made to these [105]. In keeping with other guidelines issued by the Renal Association, we have used the modified GRADE system. This grading system classifies expert recommendations as ‘strong’ (Grade 1) or ‘weak’ (Grade 2) based upon balance between the benefits and risks, burden and cost. The quality or level of evidence is designated as high (Grade A), moderate (Grade B), low (Grade C) or very low (D) depending on factors such as study design, directness of evidence and consistency of results. Grades of recommendation and quality of evidence may range from 1A to 2D [129, 210].

These guidelines are based upon systematic literature searches conducted between November 2014 and February 2016. The main searches were performed in November-December 2014 and then rerun in February 2016. We searched Pubmed/MEDLINE, the Cochrane database of systematic reviews and hand searched reference lists and articles identified by the writing group members up till March 2016. We also reviewed all related guidelines from the National Institute for Clinical Excellence, NHS Blood and Transplant, the Advisory Committee on the Safety of Blood, Tissues and Organs (SaBTO), Kidney Disease Improving Global outcomes (KDIGO), the European Renal Association Best Practice Guidelines, Caring for Australians with Renal Impairment (CARI) and American Society of Transplant Surgeons. We cross-referenced with the previous iteration of these guidelines. The Pubmed search terms used were ‘kidney transplant’ AND rejection/rejection/immunosuppression/cancer/cardiovascular/diabetes/obesity/smoking/hypertension/pregnancy/gout/infection/vaccination/fertility/pregnancy.

## Summary of clinical practice guidelines for the post-operative care of the kidney transplant recipient

### Kidney Transplant Recipient (KTR): organisation of outpatient follow-up (guidelines 1.1–1.4)

#### Guideline 1.1 – KTR: clinic infrastructure

We suggest that the following infrastructure should be in place for KTR follow up (2D):

- A consultant-level health care professional should be available for every transplant clinic
- KTRs should be reviewed in a dedicated outpatient area
- The results of blood tests (including drug levels if possible) should be available within 24 h
- A formal mechanism should exist for results review by health care professionals within 24 h of a clinic appointment
- There should be access to a multidisciplinary renal team including pharmacist, dietician, social worker and psychologist
- Patient care should be planned along principles set out in the National Service Framework and “Kidney Health Delivering Excellence”

#### Guideline 1.2 – KTR: clinic frequency

We suggest that uncomplicated patients may be reviewed progressively less frequently (2C)

- 2–3 times weekly for the first month after transplantation
- 1–2 times weekly for months 2–3
- Every 2–4 weeks for months 4–6
- Every 4–6 weeks for months 6–12
- 3–6 monthly thereafter

#### Guideline 1.3 – KTR: patient access

We suggest that all patients should have access to support services and results. (2C)

- All patients should have the option of on-line access to their results via the “Patient View” service
- All patients should have open access to the renal transplant outpatient service and have an established point of contact for enquiries
- Patient information should be available in both written and electronic formats

#### Guideline 1.4 – KTR: chronic transplant care review

We suggest that a detailed review should be performed annually post-operatively (2C)

- A process should exist for patient review on an annual basis in a different format of clinic according to the “Care plan model”
- This should be a patient-centred clinic, facilitated by a health care professional
- It should address concerns in medical, social, psychological and sexual domains
- Access to a renal dietician, social worker, specialist renal pharmacist and/or psychologist should be readily available from this clinic
- This process should proceed in parallel with formal medical review

### Kidney Transplant Recipient: Non-adherence (Guideline 2.1)

#### Guideline 2.1 – KTR: recognising non-adherence

We suggest that it is important to prevent and detect non-adherence in kidney transplant recipients. (2C)

- Factors associated with non-adherence should be identified
- An established interventional pathway should be in place for those at high risk of or with proven non-adherence
- Pathways should be in place for paediatric KTRs in transition and for adolescent KTRs

### Kidney Transplant Recipient: immunosuppressive treatment (Guidelines 3.1–3.12)

#### Guideline 3.1 – KTR: immunosuppression regimen

We recommend that the patient and/or carer should be engaged in the decisions around selection of induction agent and maintenance immunosuppression (1D)

#### Guideline 3.2 – KTR: induction immunosuppression

We recommend induction therapy should take into account the following:

- Immunosuppressive drugs should be started before or at the time of renal transplantation (1B)
- Induction therapy with biological agents should be administered to all KTRs. In patients at low immunological risk this will generally involve an interleukin-2 receptor antagonist (IL2-RA). Recipients at higher immunological risk may be considered for T-cell (lymphocyte) depleting antibodies (TDAs) (1B)
- Induction therapy with TDAs may also be useful for lower immunological risk patients with the intention of either steroid or calcineurin inhibitor (CNI) avoidance (1C)

#### Guideline 3.3 – KTR: induction immunosuppression

We suggest that a CNI should be started at the time of transplantation and not delayed until the graft is functioning (2C)

#### Guideline 3.4 – KTR: maintenance immunosuppression

We recommend that maintenance immunosuppression should normally consist of a calcineurin inhibitor (CNI) and an anti-proliferative agent, with or without corticosteroids in low and medium immunological risk KTRs (1B)

#### Guideline 3.4 – KTR: maintenance immunosuppression

We suggest that low-medium dose tacrolimus (trough target 4–8 ng/mL) is recommended as the CNI of choice in patients also taking steroids who are low and medium immunological risk and are not at high risk of developing post transplant diabetes mellitus (PTDM) (2C)

#### Guideline 3.5 – KTR: maintenance immunosuppression

We suggest that mycophenolic acid -based drugs should be the first-line anti-proliferative agent in preference to azathioprine, except in fertile KTRs who are unwilling to use reliable contraception (2B)

#### Guideline 3.5 – KTR: maintenance immunosuppression

We suggest that slow release tacrolimus may be used as an option as second line agents for patients who suffer intolerable side effects related to peak dose toxicity (2C)

#### Guideline 3.6 – KTR: maintenance immunosuppression

We suggest that KTRs who are unable to tolerate tacrolimus or who suffer serious adverse reactions related to its use be considered for the use of second line agents such as ciclosporin, sirolimus, everolimus, or belatacept (1B)

#### Guideline 3.7 – KTR: maintenance immunosuppression

We suggest that MPA-based drugs should be the first-line antiproliferative agent, in preference to azathioprine, except in fertile KTRs who are unwilling to use reliable contraception (2B)

#### Guideline 3.8 – KTR: maintenance immunosuppression

We suggest that mycophenolate mofetil (MMF) and enteric-coated mycophenolate sodium (Myfortic®) provide equivalent maintenance immunosuppression (2B)

#### Guideline 3.9 – KTR: maintenance immunosuppression

We suggest that steroid avoidance or steroid withdrawal can be used during the first week after transplantation in low immunological risk kidney transplant recipients (2B)

#### Guideline 3.10 – KTR: maintenance immunosuppression

We suggest aiming for minimum target levels for CNIs in uncomplicated renal transplantation after 3 months (2C)

#### Guideline 3.11 – KTR: maintenance immunosuppression

We suggest that CNIs should not be withdrawn (2B)

#### Guideline 3.12 – KTR: maintenance immunosuppression

We suggest that if steroids are not withdrawn within the first month, then they should be continued at low dose (prednisolone 5 mg per day or less) (2C)

#### Guideline 3.13 – KTR: monitoring of immunosuppression

We suggest that long-term monitoring of immunosuppression levels is required as follows:

- Tacrolimus and ciclosporin levels should be monitored. The initial frequency should be 3 times a week. Levels should also be checked when any medication with possible interactions is prescribed, the dosage is changed, the formulation is changed, or when there is unexplained graft dysfunction (2C)
- Tacrolimus should be monitored by the trough (C_0_) level, while ciclosporin can be monitored by either C_0_ or 2-h post dose (C_2_) level (2C)
- Tacrolimus and ciclosporin levels should be available within 24 h of taking blood samples in the first 3 months after transplantation (2D)
- The utility of monitoring mycophenolic acid (MPA) C_0_ levels is uncertain (2D)
- Sirolimus should be monitored by the C_0_ trough level (2C)

#### Guideline 3.14 – KTR: prescribing and the use of generic agents

We suggest that generic immunosuppression compounds should not be used unless they have been shown to be bioequivalent and approved by the European Agency for the Evaluation of Medicinal Products (2D)

#### Guideline 3.15 – KTR: prescribing and the use of generic agents

We suggest that KTRs should be made aware of the existence of generics and the importance of not switching between preparations without appropriate supervision (2D)

#### Guideline 3.16 – KTR: prescribing and the use of generic agents

We suggest that drugs should be prescribed by brand name (whether branded or generic drugs are prescribed) (2D)

#### Guideline 3.17 – KTR: prescribing and the use of generic agents

We suggest that KTRs should be closely monitored after switching between generic preparations until a new steady state is established (2D)

### Kidney Transplant Recipient: acute rejection (Guidelines 4.1–4.12)

#### Guideline 4.1 – KTR: diagnosis of acute rejection

We recommend that a transplant renal biopsy should be carried out before treating an acute rejection episode unless this will substantially delay treatment or pose a significant risk to the patient (1C)

#### Guideline 4.2 – KTR: diagnosis of acute rejection

We suggest that two cores of renal tissue should be obtained at transplant biopsy since this will increase the sensitivity of the investigation (2C)

#### Guideline 4.3 – KTR: diagnosis of acute rejection

We suggest that a 16 gauge automated core biopsy needle is used where possible to provide the best compromise between diagnostic usefulness and patient tolerance of the procedure (1C)

#### Guideline 4.4 – KTR: diagnosis of acute rejection

We recommend that a protocol transplant renal biopsy, defined as a biopsy performed in a stable graft without clinical evidence of acute rejection, be considered in the setting of persisting delayed graft function (1C)

#### Guideline 4.5 – KTR: diagnosis of acute rejection

We recommend that routine C4d and SV40 staining should be performed upon transplant biopsies to address other causes of graft dysfunction (2C)

#### Guideline 4.6 – KTR: diagnosis of acute rejection

We suggest that a serum sample be sent at the time of renal biopsy (for graft dysfunction) to look for human leucocyte antigen (HLA)-specific antibodies (2C)

#### Guideline 4.7 – KTR: treatment of acute rejection

We suggest that borderline acute cellular rejection should be treated in the context of acute graft dysfunction (2D)

#### Guideline 4.8 – KTR: treatment of acute rejection

We recommend that high dose intravenous corticosteroids should be the first line treatment for acute cellular rejection (1D)

#### Guideline 4.9 – KTR: treatment of acute rejection

We suggest that maintenance steroids should be added or restarted in steroid-free patients undergoing acute rejection of any type (2D)

#### Guideline 4.10 – KTR: treatment of acute rejection

We suggest that lymphocyte depleting agents may be considered for refractory acute cellular rejection or aggressive vascular cellular rejection (i.e. Banff category 4 Type II and III) (2C)

#### Guideline 4.11.1 – KTR: treatment of acute rejection

We suggest that antibody mediated rejection (AMR) should be treated with one or more of the following modalities: steroids; plasma exchange; intravenous immunoglobulin; anti-CD20 antibody, lymphocyte-depleting antibody or bortezomib (2C)

#### Guideline 4.11.2 -KTR: treatment of acute rejection

We recommend that the British Transplant Society (BTS) guidelines on antibody incompatible transplantation for management of rejection in the context of antibody incompatible transplantation (1A-D)

#### Guideline 4.12 – KTR: treatment of acute rejection

We suggest that - after an episode of rejection (unless associated with low CNI levels) - azathioprine should be switched to MPA-based immunosuppression, MPA should be started, or the existing dose of MPA maximised (2D)

### Kidney Transplant Recipient: chronic allograft injury (Guidelines 5.1–5.7)

#### Guideline 5.1 – KTR: diagnosis of chronic allograft injury

We recommend that early identification of graft injury is desirable to maximise the potential for intervention. A proactive and systematic approach should employed to manage graft dysfunction (1C)

#### Guideline 5.2 – KTR: detection of chronic allograft injury

We suggest that renal function should be monitored at each clinic visit by assessment of serum creatinine and qualitative evaluation of urine protein excretion by dipstick, supplemented by spot protein:creatinine ratio (PCR) or albumin:creatinine ratio (ACR) if positive (2C)

#### Guideline 5.3 – KTR: diagnosis of chronic allograft injury

We suggest that renal biopsy is the optimal investigation for parenchymal causes of graft dysfunction where the cause is uncertain (2C)

#### Guideline 5.4 – KTR: diagnosis of chronic allograft injury

We suggest that renal biopsies in patients with chronically deteriorating function should be stained for C4d and SV40 (2C)

#### Guideline 5.5 – KTR: diagnosis of chronic allograft injury

We suggest that a serum sample should be sent at the time of renal biopsy (for graft dysfunction) to look for HLA-specific antibodies (2C)

#### Guideline 5.6 – KTR: treatment of chronic allograft injury

We suggest that chronic allograft injury should be treated:

- By withdrawal of calcineurin inhibitors if there is histological evidence of CNI toxicity or non-specific interstitial fibrosis and tubular atrophy (2C)
- By intensification of immunosuppression if there is evidence of ongoing immune injury (cellular rejection and/or humoral rejection) (2C)
- In a similar fashion to other patients with chronic kidney disease (CKD), following similar preventative strategies and with timely referral to low clearance services (2D)

#### Guideline 5.7 – KTR: renal biopsy in chronic allograft injury

We suggest that a renal transplant biopsy is indicated:

- If there is a persistent unexplained elevation of creatinine or failure to return to baseline after an episode of biopsy proven acute rejection (BPAR) (1C)
- Every 7–10 days during delayed graft function (DGF) (2C)
- If expected renal function is not achieved within 4–8 weeks (2D)
- If sustained new onset proteinuria develops (PCR >50 mg/mmol or ACR >35 mg/mmol) (2C)

### Kidney Transplant Recipient: cardiovascular disease and lifestyle (Guidelines 6.1–6.6)

#### Guideline 6.1 – KTR: hypertension

We suggest that the management of hypertension take into account that:

- Blood pressure should be recorded at each clinic visit (1C)
- Clinic blood pressure should be <140/90 mmHg in clinic (130/80 mmHg if PCR >50 or ACR >35) (2C)
- Home blood pressure recordings and 24-h ambulatory recordings may be helpful in some instances but lower BP targets should then be set (home and or ambulatory daytime measures <135/80 mmHg) (2D)
- There is no evidence that any antihypertensive agent is better than any other and effort should be focused on achieving absolute blood pressure control rather than the use of individual agents (2D)
- Inhibitors of the renin-angiotensin system may be more effective in the minimisation of proteinuria but should be used with caution in the first 3 months post-transplant (2C)
- Resistant hypertension may be due to transplant renal artery stenosis and should be investigated according to local practice (2D)

#### Guideline 6.2 – KTR: dyslipidaemia

We suggest that the management of dyslipidaemia take into account that:

- Fasting lipid levels should be measured on an annual basis in renal transplant recipients (2C)
- Treatment targets should be the same as in the general population (2C)
- KTRs at increased primary or secondary cardiovascular risk receive statin therapy to reduce the risk of coronary artery disease (2C)
- The choice and dose of statin should take into account concurrent immunosuppression. High dose simvastatin (≥40 mg daily) should be avoided in conjunction with ciclosporin and/calcium channel antagonists (2D)

#### Guideline 6.3 – KTR: diabetes mellitus

We suggest that the detection and treatment of diabetes should consider:

- Screening for the development of post-transplant diabetes mellitus (PTDM) by dipstick urinalysis and measurement of blood sugar level at each clinic visit (2C)
- Post-transplant immunosuppression should take into account risk factors for the development of diabetes (2C)
- The diagnosis of PTDM is made based on WHO criteria for the diagnosis of diabetes mellitus based on fasting or random blood, serum glycated haemoglobin (HBA1c) or oral glucose tolerance testing (1C)
- A diagnosis of PTDM is made once patients are established on stable maintenance immunosuppression (2D)
- Post-transplant diabetes should be managed in collaboration with specialists in diabetic medicine (2D)
- All units should have a protocol for the management of post-transplant diabetes (2C)
- KTR with diabetes (either prior to transplantation or PTDM) should undergo screening for diabetic complications (retinal screening, foot care, neuropathy) in line with guidelines for non KTR patients with diabetes (2D)

#### Guideline 6.4 – KTR: ischaemic heart disease

We suggest that KTRs receive standard treatment for ischaemic heart disease, including thrombolysis, revascularisation, and secondary prevention (2C)

#### Guideline 6.5 – KTR: smoking cessation

We recommend that smoking should be strongly discouraged in transplant recipients (see guideline 6.4) (1A)

#### Guideline 6.6 – KTR: lifestyle measures

We suggest that advice on healthy lifestyle forms a routine part of post-transplant care:

- Maintenance of a healthy diet should be encouraged (2C)
- An ideal weight should be targeted (body mass index (BMI) ≤25 kg/m^2^) (2C)
- Weight management services should be available (2C)
- We suggest that KTRs participate in physical activity at a level similar to that recommended to age and co-morbidity matched counterparts from the general population (2D)
- Alcohol consumption should be within national guidelines (2D)
- Recreational drug use should be avoided (2D)
- The use of over-the-counter medications (without discussion with clinical staff) and non-proprietary medications (e.g. herbal medicines) should be discouraged (2D)

### Kidney Transplant Recipient: neoplasia (Guidelines 7.1–7.4)

#### Guideline 7.1 – KTR: screening for cancer

We suggest that the organisation of screening for neoplasia in KTRs take into account:

- Screening should be similar to the general population for cervical, breast, colon and prostate cancer (2C)
- Screening is not recommended for renal cell carcinoma (2C)
- Patient education pre and post transplantation (1C)
- Patients should be aware of malignancy risk and encouraged to report symptoms which may represent *de novo* malignancy (e.g. breast or testicular lumps) (2D)
- Skin surveillance by a healthcare professional at least biannually up to 5 years post-transplant and annually from 5 years post-transplant (2C)
- Patients with cirrhosis should undergo an annual hepatic ultrasound and determination of serum alpha feto-protein (2C)

#### Guideline 7.2 – KTR: Non-melanoma skin cancer (NMSC)

We recommend that KTRs should be educated about the adverse effects of sun exposure (1C)

#### Guideline 7.3 – KTR: Non-melanoma skin cancer

We suggest that KTRs that an individualised assessment of hazard should be made according to risk factors (2C)

#### Guideline 7.4 – KTR: Non-melanoma skin cancer

We recommend that patients should be encouraged to cover their skin in direct sunlight and to use total sunblock (Sun Protection Factor ≥50) (1D)

#### Guideline 7.5 – KTR: Non-melanoma skin cancer

We suggest that self-examination should be encouraged with guidance provided. This should be supplemented by at least biannual review by a trained healthcare professional up to 5 years post-transplant and annual review from 5 years (2C)

#### Guideline 7.6 – KTR: Non-melanoma skin cancer

We suggest that the prescription of acitretin as chemoprophylaxis be considered in those with ≥2 previous NMSC if there are no contraindications (2B)

#### Guideline 7.7 – KTR: immunosuppression in cancers

We suggest that immunosuppression should be reduced if neoplasia develops (2C)

#### Guideline 7.8 – KTR: immunosuppression in cancers

We suggest that mammalian target of rapamycin inhibitors (m-TORi) are considered as alternative immunosuppressive agents in KTRs who develop *de novo* malignancy (2C)

#### Guideline 7.9 – KTR: immunosuppression in Kaposi sarcoma

We suggest that m-TORs have specific anti-tumour effects in Kaposi sarcoma (2C)

## Kidney Transplant Recipient: infection complications (Guidelines 8.1–8.9)

### Guideline 8.1 – KTR: vaccination

#### Guideline 8.1.1 – KTR: vaccination

We recommend that KTRs:

- Should be vaccinated with inactivated viruses as per the normal population (1D)
- Should receive annual influenza vaccination unless contraindicated (1C)

#### Guideline 8.1.2 – KTR: vaccination

We suggest that KTRs:

- Should have hepatitis B surface antibody (HBsAb) levels rechecked annually and be revaccinated if antibody titres fall below 10 mIU/mL (2D)
- Should not receive live attenuated vaccines (2C)
- Should receive pneumococcal vaccine and a booster every five years (2D)

### Guideline 8.2 – KTR: cytomegalovirus (CMV) disease

#### Guideline 8.2.1 – KTR: prophylaxis and treatment of CMV disease

We recommend:

- Prophylaxis should be continued for 3–6 months, until immunosuppression has been reduced to long-term maintenance level (1B)
- Treatment should be administered for 6 weeks after treatment with a TDA (1C)

#### Guideline 8.2.2 – KTR: prophylaxis and treatment of CMV disease

We suggest:

- All transplant units should be able to measure CMV serological status and quantify viral load (2D)
- Donor and recipient CMV status should be recorded at the time of transplantation (2D)
- Each unit should have a written protocolised CMV strategy based on prophylaxis or pre-emptive therapy (2D)
- For the treatment of mild and moderate CMV disease, oral valganciclovir and intravenous ganciclovir are of equivalent efficacy (2C)
- The first line treatment of life-threatening CMV disease is intravenous ganciclovir (2D)
- Treatment duration should be determined by monitoring viral load (2C)

### Guideline 8.3 – KTR: Epstein Barr Virus (EBV) infection

#### Guideline 8.3.1 – KTR: EBV infection

We recommend that immunosuppression should be reduced or stopped following the development of post transplant lymphoproliferative disease (PTLD) (1C)

#### Guideline 8.3.2 – KTR: EBV infection

We suggest:

- Both donor and recipient should have their EBV serology recorded at the time of transplantation (2D)
- All high risk (D^+^/R^−^) patients (including adults) should have EBV viral load measured immediately after transplantation, monthly for 6 months, and 3 monthly to 1 year (2C)
- EBV viral load should be monitored after the treatment of rejection (2C)
- Total immunosuppression should be reduced when EBV titres rise significantly (2C)

### Guideline 8.4 – KTR: Varicella Zoster Virus (VZV) infection

#### Guideline 8.4.1 – KTR: VZV infection

We recommend:

- Primary infection (chickenpox) should be treated with intravenous aciclovir or oral valaciclovir until the lesions scab over (1C)
- Uncomplicated shingles should be treated with oral acyclovir or valaciclovir until the lesions scab over (1D)
- Disseminated (>2 dermatomes), ocular or invasive shingles should be treated with intravenous aciclovir until the lesions scab over, together with a reduction in immunosuppression (1B)
- Varicella-susceptible KTRs (i.e. VZV IgG negative) with primary exposure to VZV should receive intravenous immunoglobulin, ideally within 96 hours, but up to a maximum of 10 days following exposure. If unavailable or after 10 days, oral aciclovir should be prescribed for seven days (1D)

#### Guideline 8.4.2 – KTR: VZV infection

We suggest:

- Patients on the waiting list who are VZV IgG negative should be vaccinated prior to transplantation (2D)
- Immunosuppression should be reduced during primary infection (2D)

### Guideline 8.5 – KTR: Herpes Simplex Virus (HSV) infection

#### Guideline 8.5.1 – KTR: HSV infection

We recommend:

- Superficial HSV infection should be treated with appropriate oral agents until the lesions have resolved (1D)
- Systemic HSV infections should be treated with intravenous aciclovir and a reduction in immunosuppression until a response occurs and oral medication should be continued for at least 14 days (1C)

#### Guideline 8.5.2 – KTR: HSV infection

We suggest that KTRs suffering frequent recurrent HSV infection should consider oral prophylaxis (2D)

### Guideline 8.6 – KTR: BK virus (BKV) nephropathy

#### Guideline 8.6.1 – KTR: BK nephropathy

We recommend that confirmed BK nephropathy should be treated by reduction in immunosuppression (1D)

#### Guideline 8.6.2 – KTR: BK nephropathy

We suggest:

- Screening should also be carried out when renal function deteriorates in an unexplained fashion (2D)
- KTRs should be screened for BKV viral load or by performing urine microscopy for decoy cells or by polymerase chain reaction (PCR) on urine or serum (2C)
- Suspected BK nephropathy should be confirmed by renal biopsy, which should be stained for SV40. Two cores containing medullary tissue should ideally be examined (2D)
- Immunosuppression should be reduced when the serum BKV load exceeds 10^4^ copies/ml (2C)
- There is no established specific treatment for BK nephropathy (2D)
- Re-transplantation can safely be considered in patients who have BK nephropathy diagnosed in an earlier graft (2C)

#### Guideline 8.7 – KTR: pneumocystis jirovecii infection - treatment and prophylaxis

We suggest:

- All patients with confirmation (microscopy or PCR) of Pneumocystis jirovecii in respiratory secretions should be treated for 14 to 21 days with co-trimoxazole orally or intravenously (15-20 mg/kg in three or four divided doses) (2B)
- Patients with contraindications to treatment with co-trimoxazole should receive pentamidine (4 mg/kg/day intravenously) (2B)
- Adjunctive glucocorticoid therapy may be considered in patients with severe disease (2D)
- All patients should receive 3–6 months of treatment with co-trimoxazole 480 mg daily for Pneumocystis jirovecii prophylaxis following renal transplantation (1B)

#### Guideline 8.8 – KTR: post-transplant infection prophylaxis

We suggest:

- All patients should receive 3–6 months of treatment with co-trimoxazole 480 mg daily (1B)
- Oral antifungal prophylaxis should be administered for 1 week after transplantation (2C)
- In selected patients, prophylaxis against mycobacterium tuberculosis with daily isoniazid (supplemented with pyridoxine) should be instituted for 6 months after transplantation (2C)

#### Guideline 8.9 – KTR: Hepatitis E Virus (HEV)

We recommend that Hepatitis E Virus (HEV)-screened blood components should be given to all KTR (1C)

### Kidney Transplant Recipient: bone and joint disease (Guidelines 9.1–9.4)

#### Guideline 9.1 – KTR: osteoporosis

We suggest:

- KTRs suffering from osteoporosis or at high potential risk should be considered for steroid-avoiding immunosuppression (2D)
- KTRs on long-term steroids or at high risk for osteoporosis should undergo DEXA scanning if eGFR >30 mL/min/1.73 m^2^ (2D)
- Treatment should be according to the Royal College of Physicians (RCP) guidelines for steroid-induced osteoporosis (2D)

#### Guideline 9.2 – KTR: tertiary hyperparathyroidism

We suggest:

- Severe hyperparathyroidism should be treated prior to transplantation (2D)
- Cinacalcet can be used in KTR (2C)
- Treatment should be the same as for other patients with CKD (2D)

### Guideline 9.3 – KTR: gout

#### Guideline 9.3.1 – KTR: treatment of gout

- We recommend that neither allopurinol nor febuxostat should be administered with azathioprine (1C)

#### Guideline 9.3.2 – KTR: treatment of gout

We suggest:

- Hyperuricaemia should be treated when associated with gout, tophi or uric acid stones (2D)
- Non-steroidal anti-inflammatory drugs (NSAIDs) should be avoided in KTRs (2D)
- Acute gout may be treated with brief a course of oral prednisolone. (2D)
- Colchicine is an effective treatment for gout in KTR (2D)

#### Guideline 9.4 – KTR: calcineurin inhibitor bone pain

We suggest:

- Reduction or withdrawal of CNIs should be considered in KTRs with intractable bone pain (2D)
- Dihydropyridine calcium antagonists also may be beneficial (2D)

### Kidney Transplant Recipient (KTR): haematological complications (Guidelines 10.1–10.3)

#### Guideline 10.1 – KTR: anaemia

We suggest that chronic anaemia should be managed in the same way as other patients with CKD (2D)

#### Guideline 10.2 – KTR: polycythaemia

We recommend that initial treatment should be with angiotensin converting enzyme inhibitors (ACEIs) or angiotensin receptor blockers (ARBs) (1C)

#### Guideline 10.3 – KTR: polycythaemia

We suggest:

- Haemoglobin levels should be monitored at every clinic visit (2D)
- Treatment should be initiated if the haematocrit or packed cell volume exceeds 52% in men and 49% in women (2D)
- Venesection may be used in refractory cases.(2D)

### Kidney Transplant Recipient (KTR): reproductive issues (Guidelines 11.1–11.5)

#### Guideline 11.1 – KTR: conception and contraception (female)

We recommend that MPA-containing immunosuppressant drugs should be stopped prior to conception and replaced appropriately (1A)

#### Guideline 11.2 – KTR: conception and contraception (female)

We suggest:

- KTRs should wait for 1 year after transplant and have stable function before attempting conception (2C)
- Counselling regarding fertility and reproduction should be offered to female KTRs and their partners either prior to transplantation or soon afterwards (2D)
- m-TORi should be stopped prior to conception and replaced as appropriate (2D)
- Pregnancy should be jointly managed with an Obstetrics department with experience of care of KTR (2D)
- KTRs receive aspirin 75 mg daily to reduce the risk of pre-eclampsia from 12 weeks gestation until birth of the baby unless there are contraindications (2C)
- The risks and benefits of breastfeeding should be discussed (2D)
- Contraception advice should be similar to the general population (2D)

#### Guideline 11.3 – KTR: conception and contraception (male)

We recommend:

- Male KTRs are advised that MPA containing compounds have theoretical teratogenic potential in men taking these agents (1D)
- KTRs should be advised that m-TORi reduce the male sperm count and counselled accordingly. (1C)

#### Guideline 11.4 – KTR: conception and contraception (male)

We suggest:

- All immunosuppressive drugs other than m-TORi can be used in male KTRs. Advice for MPA is as Guideline 11.3 (2D)
- The decision to continue MPA containing compounds in a male KTR wishing to conceive should balance the risk of theoretical teratogenicity against the risk of rejection on changing from MPA to azathioprine (2D)
- Men on m-TORi who wish to conceive should discontinue these agents prior to conception and replace them as appropriate (2D)
- Men who wish to maintain fertility should avoid m-TORi or bank sperm prior to starting these drugs (2D)

#### Guideline 11.5 – KTR: sexual dysfunction

We suggest:

- Specific enquiry should be made regarding sexual dysfunction, preferably at an annual review clinic (2D)
- Care pathways for dealing with sexual dysfunction should be established (2D)
- Close liaison with local andrology service is recommended (2D)
- Sildenafil is safe and effective in male KTR not taking nitrates (2D)

## Summary of audit measures for the post-operative care of the kidney transplant recipient

- Proportion of blood results available for review, and reviewed, within 24 h
- Proportion of units with a written follow-up schedule available to all staff and patients
- Percentage of patients accessing their results through Renal Patient View
- Percentage of total patients assessed in an annual review clinic
- Recording “Did Not Attend” (DNA) rates for all patients
- Recording sub-therapeutic drug levels. Measuring within-patient variability of CNI levels
- Percentage of total patients receiving induction with ILRAs and TDAs
- Percentage of de novo KTRs receiving tacrolimus
- Percentage of de novo KTRs receiving MPA based immunosuppression
- Percentage of de novo KTRs receiving corticosteroid maintenance therapy
- Use of generic agents
- Severity of biopsy proven acute rejection (BPAR) recorded by Banff criteria
- Percentage of KTRs with BPAR in first 3 months and first 12 months
- Percentage of KTRs requiring TDAs to treat rejection in first year
- Complication rates after renal transplant biopsy
- The percentage of KTRs with BPAR in first 3 months and the first 12 months
- The percentage of KTRs with a donor specific HLA antibody at the time of biopsy
- The percentage of KTRs with positive C4d staining on biopsy
- Proportion of patients receiving a target blood pressure of 140/90 mmHg or 130/80 mmHg in the presence of proteinuria (PCR >100 mg/mmol or ACR >70 mg/mmol)
- Proportion of patients with proteinuria assessed by dipstix and, if present, quantified at each clinic visit
- Proportion of renal transplant recipients with an annual fasting lipid profile
- Proportion of RTR taking statins (including the type of statin) for primary and secondary prevention of premature cardiovascular disease
- Proportion of patients on other lipid lowering agents
- Proportion of patients achieving dyslipidaemia targets
- Incidence of post-transplant diabetes mellitus (PTDM) at 3 months and at annual intervals thereafter
- Proportion of patients who require insulin, and in whom remedial action is undertaken – minimisation of steroids and switching of CNIs
- The proportion of patients with PTDM enrolled in screening for extra-renal complications of PTDM
- Proportion of patients with ischaemic heart disease
- Proportion of patients suffering myocardial infarction
- Proportion of patients undergoing primary revascularisation
- Proportion of patients receiving secondary prevention with a statin, anti-platelet agents and renin angiotensin system (RAS) blockers
- Proportion of KTRs who smoke
- Proportion of cigarette smoking KTRs who have been given formal advice or offered help with cessation
- Proportion of patients who are obese (BMI > 30 kg/m^2^)
- Proportion of patients having screening procedures for neoplasia at the annual review clinic
- Incidence of CMV disease
- Rate of EBV infection and PTLD
- Completeness of records for EBV donor and recipient serology
- Rates of primary VZV and shingles infection
- Completeness of records for VZV recipient serology
- Rates and outcomes of HSV infections
- Rates of BK viral infection in screening tests
- Rates and outcomes of BK nephropathy
- Frequency of bisphosphonate use
- Incidence of fractures
- Incidence of hyperparathyroidism
- Incidence of parathyroidectomy
- Use of cinacalcet
- Frequency of hyperuricaemia and gout
- Prevalence of anaemia
- Prevalence of polycythaemia
- Pregnancy rates and outcomes
- Prevalence of sexual dysfunction

## Rationale for clinical practice guidelines for post-operative care of the kidney transplant recipient

### Kidney Transplant Recipient (KTR): organisation of outpatient follow-up (Guidelines 1.1 – 1.4)

#### Guideline 1.1 – KTR: clinic infrastructure

We suggest that the following infrastructure should be in place for KTR follow up (2D)

- A consultant-level health care professional should be available for every transplant clinic
- KTRs should be reviewed in a dedicated outpatient area
- The results of blood tests (including drug levels if possible) should be available within 24 h.
- A formal mechanism should exist for results review by health care professionals within 24 h of a clinic appointment
- There should be access to a multidisciplinary renal team including pharmacist, dietician, social worker and psychologist
- Patient care should be planned along principles set out in the National Service Framework and “Kidney Health Delivering Excellence”

##### Audit Measure

The proportion of blood results available for review, and reviewed, within 24 h

##### Rationale

All KTRs should have ready access to a senior clinical opinion and a senior clinician should be available at renal transplant clinics. In some centres this may be a consultant-level nurse, in others a medical or surgical consultant. The exact type of healthcare professional is not important but KTRs and junior staff should have access to an individual with appropriate knowledge and experience. This will also benefit the training of junior medical staff. A dedicated outpatient area is beneficial as it provides a familiar environment and staff experienced in the management of patients on renal replacement therapy.

Prompt availability and formal review of test results is desirable since most complications can be resolved more easily if recognised at an early stage, particularly in the first few weeks after renal transplantation. It is recommended that patient care is carried out according to the principles laid out in the Department of Health (DoH) leaflet, “Achieving Excellence in Kidney Care [47] and the report by Kidney Research UK “Kidney Health Delivering Excellence” [211].

#### Guideline 1.2 – KTR: clinic frequency

We suggest that uncomplicated patients, in genral, may be reviewed progressively less frequently in clinic (2C)

- 2–3 times weekly for the first month after transplantation
- 1–2 times weekly for months 2–3
- Every 2–4 weeks for months 4–6
- Every 4–6 weeks for months 6–12
- 3–6 monthly thereafter

##### Audit Measure

Proportion of units with a written follow-up schedule available to all staff and patients

##### Rationale

Freedom from regular hospital attendance is an important benefit of renal transplantation, balanced against the risks and prevention of complications. These risks (especially of surgical complications) are highest in the immediate postoperative period and during the first few weeks following hospital discharge, when the burden of immunosuppression is greatest. For typical patients monitoring should therefore be most frequent during this period and then diminish with time. The use of virtual renal clinics should be explored as a complementary form of KTR review as it might be more convenient for some patients.

#### Guideline 1.3 – KTR: patient access

We suggest that all patients should have ready access to support services and results (2C)

- All patients should have on-line access to their results via the “Renal Patient View” service if they wish
- All patients should have open access to the renal transplant outpatient service and have an established point of contact for enquiries
- Patient information should be available in both written and electronic formats

##### Audit Measure

Percentage of patients accessing their results through Renal Patient View

##### Rationale

Patients should be encouraged to take an active role in their own care according to principles embodied in the National Service Framework [47]. Interest in their own blood results should be welcomed and KTRs should be encouraged to use Patient View (https://www.patientview.org/#/; formerly known as Renal Patient View). Patient education is a crucial element in the success of renal transplantation and easy access to information should be provided for all patients in different formats (e.g. paper-based and electronic).

#### Guideline 1.4 – KTR: chronic transplant care review

We suggest that a detailed review should be performed annually post-operatively (2C)

- A process should exist for patient review on an annual basis in a different format of clinic according to the “Care plan model”
- This should be a patient-centred clinic, facilitated by a health care professional
- It should address concerns in medical, social, psychological and sexual domains
- Open access to a renal dietician, social worker, specialist renal pharmacist and/or psychologist should be readily available from this clinic
- This process should proceed in parallel with formal medical review

##### Audit Measure

Percentage of total patients assessed in an annual review clinic

##### Rationale

Since KTRs experience considerable late morbidity which is unlikely to be managed properly in a traditional clinical setting (e.g. skin lesions, sexual dysfunction and psychological morbidity) it seems sensible to facilitate periodic follow up in a different and more holistic environment [173, 193].

### Kidney Transplant Recipient (KTR): Non-adherence (Guideline 2.1)

#### Guideline 2.1 – KTR: recognising non-adherence

We suggest that it is important to prevent and detect non-adherence in kidney transplant recipients (2C)

- Factors associated with non-adherence should be identified
- An established interventional pathway should be in place for those at high risk of or with proven non-adherence
- Pathways should be in place for paediatric KTRs in transition and for adolescent KTRs

##### Audit Measures

- Recording “Did Not Attend” (DNA) rates for all patients
- Recording sub-therapeutic drug levels
- Measuring within-patient variability of CNI levels

##### Rationale

Non-adherence with immunosuppressive medication is an important factor in graft loss and up to a third of patients report regularly missing tablets [28, 153, 166, 206]. One Dutch study described self-reported non-adherence rates of 17% at 6 weeks after transplantation rising to 27% by 6 months [134]. Clinical parameters associated with non-adherence are well recognised and should be used to assessing risk e.g. erratic or low immunosuppression levels, clinic non-attendance, psychiatric illness, low belief in the need for medication, adolescence and early adulthood [153, 166, 206]. The patient and/or their carer should be fully engaged in identifying reasons for and strategies to address non-adherence. High within-patient variability of CNI levels has also been shown to be associated with poor graft outcomes and can readily be monitored in the clinic [99, 184, 214]. It remains to be proven whether there is a prospective intervention in such patients that can improve outcomes.

### Kidney Transplant Recipient (KTR): immunosuppressive treatment (Guidelines 3.1 – 3.17)

#### General concepts

The starting point for renal transplantation is comparison with other forms of renal replacement therapy (RRT). Renal transplantation provides a better quality of life, an increased sense of well-being and a longer life span when compared to other forms of RRT. Therefore minor differences in clinical outcome between different immunosuppressive regimes should be placed in context with the much greater difference in outcome between transplantation and other forms of RRT, for those fit enough to be wait listed (approximately 30% of those with end stage renal disease).

Almost all renal transplants are allogeneic (i.e. not from identical twins) and will provoke a powerful immunological rejection response in the recipient. Rejection will destroy renal tissue and so the primary aim of immunosuppression is to avoid rejection. In general, more potent immunosuppressive regimes will reduce the risk of all forms of rejection but at the expense of increased side effects. Side effects comprise generic immunosuppressive side effects (e.g. increased risk of infection and malignancy) or those specific to the particular drug used (e.g. gingival hypertrophy with ciclosporin).

Immunosuppressive management may be divided into three phases – induction, early (<3–6 months post-transplant), and late (>3–6 months). More intensive immunosuppression is required in the early post-operative period to prevent acute rejection episodes, while long-term immunosuppression should balance the risk of rejection against the adverse effects of immunosuppressive therapy. Effective immunosuppression is best achieved by combination therapy that minimises the side effects of individual agents. Overall, the aim of immunosuppression is to maximise patient and graft survival following transplantation and to maximise the quality of life and economic benefits of transplantation.

When planning immunosuppressive treatment, it is essential to consider the risks to the recipient. The risks of immunosuppressive therapy are largely predictable and should be balanced against the risk of harm to the individual patient from under-immunosuppression and resulting rejection, and the benefits of a well-functioning transplant. The assessment of risk is imprecise but Table 1 illustrates some broad principles. Many transplant units employ such risk stratification but there is very little evidence to support such an approach since most studies have excluded high-risk patients.

**Table 1:** Risk stratification for selection of immunosuppression in kidney transplantation

Risk Type	Low	Medium	High	Possible Strategy
Immunological	0-DR mismatch First graft Unsensitised Recipient >60	1-DR mismatch Afro-Caribbean recipient Historical DSAs NDSAs DGF Older donor [45]	2-DR mismatch Previous early immunological graft loss DSAs ABO-incompatible Sensitised (high CRF/PRA) Preoperative anti-ATIIR Abs [117]	Increase total immunosuppressive load
Metabolic	Low BMI Age <40 Normal pre-Tx GTT	Positive family history ADPKD	Impaired GT BMI >35 HCV positive Age >60 Previous CVD Race	Avoid/minimise steroids and tacrolimus
Neoplastic	Age <40	Pre-malignant lesion	Previous cancer Hereditary syndrome e.g. VHL	Consider low immunosuppression load or sirolimus
Ischaemia-reperfusion injury	Living donor Deceased donor <40	CIT >12 h Donor aged 50–60	DCD CIT > 24 h Extended criteria donor	Reduce CNI exposure
Non-adherence			Poor RRT compliance Age <20 Transition from paediatric to adult	Education Simple drug regime alemtuzumab or belatacept

For the purpose of these guidelines, immunosuppression has been broadly divided into induction and maintenance phases; the maintenance phase can be further divided into early and late. While the distinction between these periods is largely arbitrary, here the induction period is considered as the peri-transplant period, the early maintenance period is the 3–6 month period after transplantation when immunosuppression is tapered, and the late maintenance phase is the period beyond 3–6 months when immunosuppression has been tapered to long-term levels. It is recognised that the renal allograft is more immunogenic during the early post-transplant period and that more potent immunosuppression is therefore required to prevent rejection. In the later maintenance phase the allograft becomes less immunogenic and more consideration can be given to the minimisation of side effects from immunosuppression.

Immunosuppressive strategies may be pre-emptive or reactive. For example, steroid avoidance is pursued by some units with the objective of avoiding steroid-related side effects. It also permits the widespread usage of tacrolimus with a reduced risk of post-transplant diabetes mellitus (PTDM). Other units adopt a strategy of dual or lower dose triple immunosuppressive drug therapy.

When considering the published evidence, it is essential to look at long-term outcome data. However, long-term data from adequately powered clinical trials are frequently not available and the best evidence comes from large registries with their inherent limitations of data collection and bias. It is also important to focus on intention-to-treat analysis to limit the bias associated with intolerance of therapy, which is common in this population.

#### Guideline 3.1 – KTR: immunosuppression regimen

We recommend that the patient and/or carer should be engaged in the decisions around selection of induction agent and maintenance immunosuppression (1D)

##### Rationale

Immunosuppressive therapy for kidney transplantation has a wide and potentially serious side effect profile, including increased risk of cancer, cardiovascular disease and infection. Engagement with patients about anticipated short and long-term side effects of immunosuppressive therapy allows discussions to reassure about some anticipated side effects, whilst informing about others. This may aid adherence with therapy and allay untoward concerns about medication side effects.

#### Guideline 3.1 – KTR: induction immunosuppression

We recommend induction therapy should take into account the following:

- Immunosuppressive drugs should be started before or at the time of renal transplantation (1B)
- Induction therapy with biological agents should be administered to all KTRs (1B). In patients at low immunological risk this will generally involve an interleukin-2 receptor antagonist (IL2-RA). Recipients at higher immunological risk may be considered for T-cell (lymphocyte) Depleting Antibodies (TDAs; e.g. anti-lymphocyte preparations [antithymocyte globulin (ATG) or alemtuzumab])
- Induction therapy with TDAs may also be useful for lower immunological risk patients with the intention of either steroid or CNI avoidance (1C)

#### Guideline 3.2 – KTR: induction immunosuppression

We suggest that a CNI should be started at the time of transplantation and not delayed until the graft is functioning (2C)

##### Audit Measure

Percentage of total patients receiving induction with ILRAs and TDAs

##### Rationale

Following allogeneic renal transplant there is an intense period of immunological activity whereby recipient lymphocytes respond to allogeneic material. Induction therapy aims to minimise this response and the risk of early graft rejection at a time when oral agents may not have reached effective concentrations.

There is good evidence that IL2-RAs reduce the risk of early rejection when compared to placebo, although there is no definitive evidence of improved graft survival at 3 years, nor are there trials of adequate statistical power to answer the question of long-term benefits. There is, however, some evidence from registry data to suggest that the lower rejection rates might translate into better graft survival [121]. Pharmacoeconomic analysis has shown that these agents are cost effective in the early post-transplant period, and this is embodied in National Institute for Clinical Excellence (NICE) guidelines (https://www.nice.org.uk/guidance/ta85) [165].

There is moderate evidence that TDAs reduce the risk of acute rejection in high-risk immunological recipients. However, this benefit is generally gained at the expense of increased side effects - in particular, an increased incidence of malignancy, cytopenia and infection. Four randomised controlled trials comparing alemtuzumab to basiliximab in standard risk patients have consistently demonstrated reduced rates of acute rejection with alemtuzumab but longer-term outcomes are still awaited [30, 70, 73, 225]. However, alemtuzumab has been associated with increased side effects in most studies and for most patients it would seem unnecessary other than as part of a strategy to avoid other drugs, e.g. corticosteroids. Anti-thymocyte globulin has been shown to reduce the incidence of acute rejection in standard risk KTRs when compared to an IL2RA, but follow up at 10 years did not show any significant clinical differences between the two groups [120]. A multicentre prospective RCT compared ATG to IL2RA in high risk KTRs and demonstrated significantly reduced rejection rates but no significant difference in 5 year outcomes [80]. Similarly, ATG may reduce acute rejection rates in black KTRs and reduce the incidence of antibody mediated rejection (AMR) and donor-specific antibody production, but long-term evidence of benefit is lacking [21, 163].

There is limited evidence to suggest that the clinical profile of alemtuzumab differs from that of other T cell depleting antibodies, with a lower incidence of post transplant lymphoproliferative disease (PTLD) [106]. Preliminary evidence suggests that that B lymphocyte depleting antibodies, such as anti-CD20, (rituximab) are not suitable as routine induction agents [36, 128]. Rituximab may be useful as induction agent in ABO incompatible transplantation [122].

#### Guideline 3.3 – KTR: maintenance immunosuppression

We recommend that maintenance immunosuppression should normally consist of a calcineurin inhibitor (CNI), an anti-proliferative agent with or without corticosteroids in low and medium immunological risk KTRs (1B)

#### Guideline 3.4 – KTR: maintenance immunosuppression

We suggest that low-medium dose tacrolimus (trough target 4-8 ng/ml) is recommended as the CNI of choice in patients also taking steroids who are low and medium immunological risk and are not at high risk of developing post transplant diabetes mellitus (PTDM) (2C)

#### Guideline 3.5 – KTR: maintenance immunosuppression

We suggest that slow release tacrolimus preparations are used as second line agents for patients who suffer intolerable side effects related to peak dose toxicity (2C)

#### Guideline 3.6 – KTR: maintenance immunosuppression

We suggest that KTRs who are unable to tolerate tacrolimus or who suffer serious adverse reactions related to its use be considered for use of second line agents such as ciclosporin, sirolimus, everolimus, or belatacept (1B)

#### Guideline 3.7 – KTR: maintenance immunosuppression

We suggest that MPA-based drugs should be the first-line antiproliferative agent, in preference to azathioprine except in fertile KTRs who are unwilling to use reliable contraception (2B)

#### Guideline 3.8 – KTR: maintenance immunosuppression

We suggest that mycophenolate mofetil (MMF) and enteric-coated mycophenolate sodium (Myfortic®) provide equivalent maintenance immunosuppression (2B)

#### Guideline 3.9 – KTR: maintenance immunosuppression

We suggest that vigilant steroid avoidance or steroid withdrawal during the first week after transplantation can generally be used in low immunological risk kidney transplant recipients (2B)

#### Guideline 3.10 – KTR: maintenance immunosuppression

We suggest that minimum target levels for CNIs should be instituted in uncomplicated renal transplantation after 3 months (2C)

#### Guideline 3.11 – KTR: maintenance immunosuppression

We suggest that CNIs should be continued rather than withdrawn (2B)

#### Guideline 3.12 – KTR: maintenance immunosuppression

We suggest if steroids are not withdrawn within the first month then they should be maintained at low dose (Prednisolone - 5 mg per day or less) (2C)

##### Audit Measures

- Percentage of *de novo* KTRs receiving tacrolimus
- Percentage of *de novo* KTRs receiving MPA-based immunosuppression
- Percentage of *de novo* KTRs receiving corticosteroid as part of maintenance therapy

##### Rationale

Immunosuppressive drugs are generally used in combination to balance effective total immunosuppression with minimisation of drug-specific side effects. Since the graft is most immunogenic in the early post-transplant period it is important to use higher doses of these drugs during this period. Thereafter dosages and thus blood levels can be reduced to minimise the risks of infection and malignancy. Account should be taken of the immunological risk of the transplant and also the strength of induction therapy, i.e. KTRs induced with TDAs often require less intensive maintenance therapy. High, medium and low trough (C_0_) levels for tacrolimus are >10, 5–10 and <5 ng/mL respectively. Comparable C_0_ levels for ciclosporin are >200, 100–200 and <100 ng/ml respectively.

It should be acknowledged that the optimal combination of immunosuppressive agents to obtain the best long-term outcomes, defined by hard endpoints such as graft and patient survival, remains elusive. There are a number of reasons for this:

- short duration of increasingly expensive trials
- convergence of outcomes with many different regimes for short term surrogate endpoints (e.g. rejection rates and graft function)
- restrictions on trial recruitment which exclude many real world patients

In the absence of such data a flexible approach is required whereby KTRs are stratified by risk and the majority are started on standard protocols. However, clinicians must be vigilant and be willing to substitute alternative agents when necessary.

Except for transplants from syngeneic or haploidentical live donors, it is generally established practice in renal transplantation to use triple therapy as maintenance, consisting of a CNI, an antiproliferative agent and corticosteroids. Such regimes have led to the lowest rejection rates and are considered the benchmark to which other regimes are compared. Induction therapy plus low-dose tacrolimus, mycophenolate mofetil (MMF) and corticosteroids have produced the lowest rates of acute rejection, superior graft function, and better graft survival. There are examples of other regimes for maintenance therapy, generally involving avoidance, minimisation or withdrawal of either steroids or CNIs. Such regimes that can produce similar outcomes in selected patients but there are currently no reliable instruments to predict which KTRs will benefit.

A large randomised controlled trial (RCT) suggested that low dose tacrolimus combined with MMF and steroids with an IL2-RA as induction was superior at 12 months in terms of graft function, graft survival and acute rejection rate to either standard or low dose ciclosporin in low immunological risk KTRs [53, 54]. There are concerns over the early nephrotoxic effects of CNIs but whether these observations extend to lower doses and levels is unknown. To date, no alternative to CNIs has been shown to improve either early or late graft outcomes. Favourable outcomes in this trial [53] were based on tacrolimus levels of 3–7 ng/mL. More recent data suggest that trough tacrolimus levels <4.0 ng/mL was associated with higher levels of rejection in the ‘post-SYMPHONY’ era [65]. Therefore a pragmatic compromise would be to aim for trough tacrolimus levels of 4-8 ng/mL.

The risk of acute rejection is minimised by early achievement of target CNI levels and so there is no reason to delay the initiation of a CNI. Specifically there is no evidence that delaying the introduction of a CNI prevents or ameliorates delayed graft function.

Trial evidence demonstrates that tacrolimus reduces the risk of acute rejection and improves graft survival during the first year of transplantation compared to ciclosporin [221]. Protocol biopsy studies also suggest that subclinical rejection is less prevalent in regimes containing tacrolimus as opposed to ciclosporin [179]. However, PTDM is significantly more common with tacrolimus even accounting for variation in concomitant steroid usage [230]. An RCT comparing tacrolimus with ciclosporin (Neoral) in non-diabetic patients demonstrated significantly higher levels of abnormalities in glucose metabolism with tacrolimus but a non-significant trend towards worse graft function with ciclosporin [216]. However, lower blood levels of tacrolimus minimise the risk of PTDM compared to higher levels; this has not been fully explored in a trial setting.

It is acknowledged that tacrolimus is associated with a number of side effects. Some milder side effects relating to peak levels of the parent compound (e.g. tremor) may be ameliorated by dose reduction or the use of slow release formulations of tacrolimus [116]. However, tacrolimus may cause disabling side effects in a minority of patients including posterior reversible encephalopathy syndrome, haemolytic uraemic syndrome, alopecia and GI disturbance. In such circumstances it is usually necessary to switch to a second line agent such as ciclosporin, m-TORi or belatacept.

Compared with placebo and azathioprine, mycophenolic acid based compounds, (MMF, mycophenolate sodium, generic MPA) reduces the risk of acute rejection [145, 189]. The evidence comparing MPA to placebo consistently demonstrates lower rates of acute rejection with MPA but at the expense of increased bone marrow suppression and increased opportunistic infection rates. Systemic review of the relevant studies suggests significantly reduced rejection rates and improved graft survival with MPA compared to azathipoprine [219]. Absolute numbers of patients with gastrointestinal side effects are higher with MMF though this is not significant. There is limited evidence that mycophenolate sodium (Myfortic) leads to a reduced incidence of gastrointestinal side effects compared to mycophenolate mofetil, albeit in studies specifically addressing patients with gastrointestinal side effects, rather that larger studies of KTRs [191]. All mycophenolic acid based compounds are associated with significant teratogenicity in both women and men and should not be used in fertile KTRs who are unwilling to use reliable contraception [146].

Steroids have a well-documented adverse effect profile and there is heightened interest in steroid withdrawal and avoidance regimes. Whether low dose prednisolone (e.g. 5 mg daily) is associated with a similar adverse profile to higher dose regimens is unknown. Interestingly there is little evidence of an effect on insulin sensitivity between no steroids and 5 mg daily [140]. The majority of accumulated trial evidence in renal transplantation has involved steroid-containing regimes and there is little information re steroid withdrawal/avoidance. There are no differences in graft survival between patients treated with or without maintenance corticosteroids beyond the first week after kidney transplantation and avoidance beyond the first week after kidney transplantation reduces adverse effects. Early withdrawal and avoidance studies show increased acute rejection rates but without an effect on graft survival [136, 137, 229]. In contrast, steroid withdrawal studies later than one month after transplantation generally show increased rejection rates. Long-term follow up is required to fully assess these effects. It is clear that close vigilance is required with steroid avoidance regimes since acute rejection rates will probably be higher. Patients with graft rejection should probably be maintained on long-term oral steroids [88].

Higher doses of CNIs are required during the first 3 months when the recipient’s immune response is receiving the most allostimulation. There is theoretically a good reason to reduce the immunosuppressive load after this time to reduce the incidence of drug-related adverse effects (i.e. reduce CNI target levels). Analysis of RCTs has shown that CNI withdrawal leads to higher rejection rates without any improvement in graft survival. Comparison of lower dose CNI regimes with higher doses have generally shown little difference in outcomes [107] but in some cases better renal function has been attained. Those seeking a fuller discussion of these studies are referred to the 2009 KDIGO guidelines [104].

While there is some evidence that m-TORis can allow reduced doses of CNIs and better graft function at 1 year after transplantation, these agents are poorly tolerated and are associated with higher rejection rates [222]. Two studies have investigated the substitution of m-TORis for CNIs between 1 and 6 months after transplantation. While both studies demonstrated short-term improvements in renal function, there were no significant improvements in long term graft or patient outcomes [26, 27, 224]. A systemic review demonstrated a significant reduction in the rate of malignancy with sirolimus but a 43% increase in overall mortality rate compared to controls [108]. The exact role of m-TORis in the early stages after transplantation is uncertain but at this time they should only be used as second line agents.

Belatacept is a fusion protein that blocks costimulatory pathways involved in T cell activation, which has to be administered by regular intravenous infusions. It has been used in KTRs as an alternative to ciclosporin and significant improvement in renal function and better graft histology at 1 year has been demonstrated [215]. More recent studies have confirmed improved graft function, which was sustained at 5 years, but also an association with the development of PTLD [177]. A meta-analysis concluded that belatacept has no demonstrable effect on rejection rates, patient or graft survival [135]. However, it was associated with significantly better graft function and histology. It is currently a valuable second line agent that may be particularly useful in certain clinical situations (e.g. poor adherence, haemolytic uraemic syndrome). In 2011, the US Food and drug administration issued a warning over risks of PTLD and progressive multifocal leukoencephalopathy (PML) with belatacept [152].

#### Guideline 3.13 – KTR: monitoring of immunosuppression

We suggest that long-term monitoring of immunosuppression levels is required as follows:

- Tacrolimus and ciclosporin levels should be monitored. The frequency should be 3 times a week immediately after the transplant. Levels should checked when any medication with possible interactions is prescribed or when there is unexplained graft dysfunction (2C)
- Tacrolimus should be monitored by the C_0_ trough level, while ciclosporin can be monitored by either trough (C_0_) or 2-h post dose (C_2_) level (2C)
- Tacrolimus and ciclosporin levels should be available within 24 h of taking blood samples (2C)
- The utility of monitoring mycophenolic acid (MPA) C_0_ levels is uncertain (2D)
- Sirolimus should be monitored by the C_0_ trough level (2C)

##### Rationale

Therapeutic drug monitoring is advisable for drugs with a narrow therapeutic index. For tacrolimus and ciclosporin the absorption may vary in the early stages after transplantation but usually stabilises within a month. Both drugs may exhibit both inter-patient and intra-patient variability. Tacrolimus and ciclosporin are traditionally monitored by 12-h C_0_ trough levels, but in the case of ciclosporin there is some evidence that C_2_ levels may also be used although target ranges are less well established and the logistics of sample collection are more complex. There is little evidence directly comparing different target levels of the same drug in a controlled fashion. Drug monitoring of MMF is best carried out by measuring the area under the curve (AUC) but clinical studies have not been conclusive [107, 114, 213]. C0 levels correlate poorly with AUC and remain unproven and rarely used in clinical practice. Sirolimus levels should be monitored since toxic effects correlate with high drug levels and C_0_ levels correlate well with AUC [98, 111].

#### Guideline 3.14 – KTR: prescribing and the use of generic agents

We suggest that generic immunosuppression compounds should not be used unless they have been shown to be bioequivalent and approved by the European Agency for the Evaluation of Medicinal Products (2D)

#### Guideline 3.15 – KTR: prescribing and the use of generic agents

We suggest that KTRs should be made aware of the existence of generics and the dangers of indiscriminate usage (2D)

#### Guideline 3.16 – KTR: prescribing and the use of generic agents

We suggest that drugs should be prescribed by brand name where unproven generic substitutes are available (2D)

#### Guideline 3.17 – KTR: prescribing and the use of generic agents

We suggest that KTRs should be followed closely after switching to a generic preparation until a new steady state is established (2D)

##### Audit Measure

- The use of generic agents should be monitored and audited

##### Rationale

The introduction of many generic preparations of tacrolimus, ciclosporin and MPA has occurred in the last 5 years. These offer potential cost savings but with the risk that these medications are not truly bioequivalent due to the limitations of the regulatory process. This has led to differences in both pharmacokinetic and clinical terms, compared with the original agents.

Immunosuppressive drugs in common use have narrow therapeutic windows, with significant risk of under- and over-immunosuppression and, with CNIs, the risk of nephrotoxicity due to over-exposure. Plasma CNI levels are carefully measured in practice, with narrow therapeutic ranges. Assessment of generic agents requires only that time-averaged plasma concentrations (area-under-the-curve) fall between 80 and 125% of the original preparation in normal subjects. Differences in bioavailability due to food, or other factors, are not assessed. For ciclosporin the bioavailability of generic agents extends across this range and is influenced by food, thus switching between generic agents may result in major differences in drug exposure. This may be minimised by careful measure of drug exposure after switching between agents and generic preparations (“named” generics). When the choice of generic is left to the dispenser this is likely to result in variable exposure. The same issues may apply to tacrolimus but the bioavailability of this agent is less variable, and to other generic immunosuppressants such as MPA, although monitoring of this agent is not usually undertaken in clinical practice. For these reasons a local protocol for the use of generic agents should be available to all involved in the care of transplant recipients, specifically those who write and dispense prescriptions.

### Kidney Transplant Recipient (KTR): acute rejection (Guidelines 4.1-4.12)

#### Guideline 4.1 – KTR: diagnosis of acute rejection

We recommend that a transplant renal biopsy should be carried out before treating an acute rejection episode unless this will substantially delay treatment or pose a significant risk to the patient (1C)

#### Guideline 4.2 – KTR: diagnosis of acute rejection

We suggest that two cores of renal tissue should be obtained if possible since this will increase the sensitivity of the investigation (2C)

#### Guideline 4.3 – KTR: diagnosis of acute rejection

We recommend that a 16 gauge automated core biopsy needle is used where possible to provide the best compromise between diagnostic usefulness and patient tolerance of the procedure (1C)

#### Guideline 4.4 – KTR: diagnosis of acute rejection

We recommend that a protocol transplant renal biopsy, defined as a biopsy performed in a stable graft without clinical evidence of acute rejection (proteinuria, rising creatinine), be considered in the setting of persisting delayed graft function (DGF) (1C)

#### Guideline 4.5 – KTR: diagnosis of acute rejection

We recommend that routine C4d and SV40 staining should be performed upon transplant biopsies (2C)

#### Guideline 4.6 – KTR: diagnosis of acute rejection

We suggest that a serum sample be sent at the time of renal biopsy (for graft dysfunction) to look for HLA-specific antibodies (2C)

##### Audit measures

- Severity of biopsy proven acute rejection (BPAR) recorded by Banff criteria
- The percentage of KTRs with BPAR in first 3 months and the first 12 months
- The percentage of KTRs requiring TDAs to treat rejection within the first year
- Complication rates after renal transplant biopsy
- The percentage of KTRs with a donor-specific HLA antibody at the time of biopsy

##### Rationale

Historically, unresolved acute rejection episodes invariably led to graft loss so it is rational to treat such episodes unless the treatment is likely to do more harm than good. Rejection episodes are characteristically associated with loss of graft function but diagnosis is best established by a percutaneous biopsy since it differentiates rejection clearly from other causes of graft dysfunction. Recognition of different forms of rejection may inform different treatment regimens (e.g. antibody mediated rejection).

Two cores of tissue should be obtained as this approach establishes the diagnosis of acute rejection with a sensitivity of 99% versus 91% when only one core is obtained [39]. The use of a 16 gauge automated core biopsy needle yields higher numbers of glomeruli and thus increased diagnostic usefulness without an increase in complication rate compared with an 18 gauge needle [149, 161].

Biopsy is performed when there is acute graft dysfunction with the aim of providing a histological diagnosis to confirm clinical suspicion. However, renal scarring may be too advanced for any intervention to lead to significant improvement. Subclinical acute rejection [185] is defined as histological rejection in the absence of clinical evidence of altered graft function diagnosed on protocol biopsies and logically one might expect that early detection would lead to improved outcomes. In most patients, with modern immunosuppression regimes, there is little evidence that treatment of SCAR improves outcomes and a clear link between SCAR and chronic rejection has not yet been proven [180]. There are multiple reasons for this: there is no large multi-centre prospective study; the induction, baseline and maintenance immunosuppression regimes vary in studies; some protocol biopsies are actually indication biopsies and thus cannot show SCAR; and the Banff criteria was designed for diagnostic biopsies and not for protocol biopsies where the findings may not fit with the criteria [71]. There is randomised control trial evidence that there is no benefit from protocol biopsies performed within the first 6 months of transplantation in low risk patients on a standard immunosuppressive regimen [180]. There are two clinical settings where protocol biopsy is of value. In delayed graft function (DGF) there is increased risk of graft failure; ‘silent’ acute rejection may account for a significant proportion of this increased risk [167]. In a more specialised setting, in very high and high immunological risk patients, if SCAR is detected this should be treated as it may improve outcome [112, 204]. The optimal timing and frequency of protocol biopsy is not clear but we suggest that it be considered in the clinical settings outlined above at days 7–10 for DGF. In high-risk transplants, protocol biopsy will typically be at 3 months.

It is recommended to stain all biopsies for C4d and to send a serum sample for detection of human leukocyte antigen (HLA) specific antibodies to facilitate diagnosis of acute antibody mediated rejection in line with joint BSHI/BTS guidelines and those of The Transplantation Society [22, 204]. The Banff criteria were revised in 2013 to acknowledge the occurrence of antibody mediated rejection in the absence of detectable C4d staining [71]. Immunohistopathologic evidence of recent interaction of antibody with the vascular endothelium is necessary for the diagnosis and this may include, but is not limited, to C4d positivity. Conversely, diffuse C4d staining may occur in the absence of morphological evidence of active rejection or graft dysfunction, primarily in ABO incompatible transplantation.

An episode of acute rejection is a period of uncertainty and is likely to cause anxiety in KTR and/or their carers. Good communication explaining the treatment rationale and likely outcomes of rejection is important in addressing these concerns.

#### Guideline 4.7 – KTR: treatment of acute rejection

We suggest that borderline acute cellular rejection should be treated in the context of acute graft dysfunction (2D)

#### Guideline 4.8 – KTR: treatment of acute rejection

We recommend that high dose intravenous corticosteroids should be the first line treatment for acute cellular rejection (1D)

#### Guideline 4.9 – KTR: treatment of acute rejection

We suggest that maintenance steroids should be added or restarted in steroid-free patients undergoing acute rejection of any type (2D)

#### Guideline 4.10 – KTR: treatment of acute rejection

We suggest that lymphocyte depleting agents may be considered for refractory acute cellular rejection or aggressive vascular cellular rejection (i.e. Banff category 4 Type II and III) (2C)

#### Guideline 4.11.1 – KTR: treatment of acute rejection

We suggest that antibody mediated rejection (AMR) should be treated with one or more of the following modalities: steroids; plasma exchange; intravenous immunoglobulin; anti-CD20 antibody; lymphocyte-depleting antibody or bortezomib (2C)

#### Guideline 4.11.2 - KTR: treatment of acute rejection

We recommend that the BTS guidelines on antibody incompatible transplantation for management of rejection in the context of antibody incompatible transplantation (1A-D)

#### Guideline 4.12 – KTR: treatment of acute rejection

We suggest after an episode of rejection (unless associated with low CNI levels) that azathioprine should be switched to MPA-based immunosuppression, MPA should be started or the existing dose of MPA maximised and ciclosporin and sirolimus should be switched to tacrolimus (2D)

##### Rationale

The management of antibody incompatible transplantation is reviewed extensively in the recent BTS guidelines on this topic [24]. In contrast to SCAR, borderline acute cellular rejection detected in the context of graft dysfunction should be treated in the knowledge that if untreated, the infiltrates may progress into rejection with consequent deterioration in transplant function [138, 231]. However, there is little evidence to guide therapy, treatment is controversial, and there is evidence that borderline infiltrates will not automatically progress into rejection [10, 231].

The majority of acute cellular rejection episodes respond to treatment with corticosteroids [66, 192]. The optimal regime for steroid administration has not been determined but intravenous methylprednisolone on three consecutive days is commonly used [66]. If intravenous steroid is precluded, high dose oral steroid can be utilised. The use of T cell depleting antibodies (TDAs) in milder grades of cellular rejection (Banff category 4 type I) may be more effective in restoring renal function but results in significantly greater side effects [196, 223]. If renal function does not return to baseline with steroid and/or ATG, or if there is a new decline in function after successful treatment of an acute rejection episode, a repeat biopsy should be considered to rule out additional rejection or other causes of graft dysfunction (e.g. concurrent acute tubular necrosis or BK nephropathy). Treating more severe cellular rejection (Banff category 4 Type IIa, IIb or III) and steroid unresponsive episodes with TDAs often improves graft function although a thorough risk-benefit assessment of such treatment should be undertaken [196, 223]. There is some evidence that adding an MPA product after such episodes or substituting azathioprine with MPA will result in fewer subsequent rejection episodes [145]. Intensifying immunosuppression after a rejection episode may help prevent further rejection including the following: maximising the dose of MPA product and switching ciclosporin or sirolimus to tacrolimus [33, 144, 202, 203].

If AMR is diagnosed, there is limited evidence that treatment with alternative modalities including plasmapheresis, immunoadsorption, intravenous immunoglobulin or monoclonal antibodies may be beneficial [176]. Trial evidence is conflicting and of low quality. Small non-randomised studies and case reports suggest that monoclonal antibodies targeting B cell function and thus antibody production (rituximab and bortezomib) may be of benefit, as may terminal complement inhibition with eculizimab. However, there is insufficient evidence to recommend these agents and the risks and benefits, particularly of biologic therapies, must be considered on an individual basis [117, 123, 176].

### Kidney Transplant Recipient (KTR): Chronic Allograft Injury (CAI) Guidelines 5.1-5.)

#### Guideline 5.1 – KTR: Diagnosis of Chronic Allograft Injury (CAI)

We recommend that early identification of graft injury is desirable to maximise the potential to intervene. A proactive and systematic approach should be employed to manage graft dysfunction (1C)

#### Guideline 5.2 – KTR: Detection of Chronic Allograft Injury (CAI)

We suggest that renal function should be monitored at each clinic visit by assessment of serum creatinine and qualitative evaluation of urine protein excretion by dipstick supplemented by spot PCR or ACR if positive (2C)

#### Guideline 5.3 – KTR: Diagnosis of Chronic Allograft Injury (CAI)

We suggest that renal biopsy is the optimal investigation for parenchymal causes of graft dysfunction where the cause is uncertain (2C)

#### Guideline 5.4 – KTR: Diagnosis of Chronic Allograft Injury (CAI)

We suggest that renal biopsies in patients with chronically deteriorating function should routinely be stained for C4d and SV40 (2C)

#### Guideline 5.5 – KTR: Diagnosis of Chronic Allograft Injury (CAI)

We suggest that a serum sample should be sent at the time of renal biopsy (for graft dysfunction) to look for HLA-specific antibodies (2C)

##### Audit Measures

- Severity of CAI recorded by BANFF criteria
- The percentage of KTRs with positive C4d staining on biopsy
- The percentage of KTRs with a donor specific HLA antibody at the time of biopsy

##### Rationale

Unfortunately there are currently no good markers of early allograft injury. A number of non-invasive biomarkers have been proposed including urine and serum microRNA profiling but the clinical utility and additive predictive value of these compared to traditional markers – serum creatinine, proteinuria, histopathology – is uncertain [5, 6, 76, 84, 132]. Graft damage can be detected by protocol biopsy but the utility of this approach is unproven. Studies show that all protocol biopsies at 3 years post transplantation display evidence of some degree of CAI and by 5 years this is classed as moderate or severe in over 60% of patients [147]. Therefore current best practice consists of vigilant monitoring of simple clinical markers of allograft function including serum creatinine and proteinuria [4, 68]. More complex and expensive approaches such as monitoring serum anti-HLA antibodies also remain unproven.

Deterioration in graft function is a heterogeneous entity with multiple causes, both immunological and non-immunological [56, 186]. Treatment may entail diametrically opposite strategies and therefore deterioration of allograft function should be investigated by percutaneous biopsy if possible. Tissue samples should be examined by an experienced renal histopathologist and classified according to the Banff criteria [71]. Staining for C4d deposition and SV40 antigen should be routinely available because positive staining will affect the treatment strategy.

Although there is not yet any proven therapy, it is important to recognise chronic humoral rejection diagnosed according to the Banff criteria [71, 142, 196]. The detection of anti-HLA antibodies and C4d staining on transplant biopsy are associated with worse clinical outcomes [16, 60, 81, 102, 115, 133, 186, 226]. More recently the complement binding ability of anti-HLA antibodies has been shown to impact upon graft survival [124]. Post-transplant screening for anti-HLA antibodies has been suggested but there is not yet a solid evidence base to recommend it routinely in low or moderate immunological risk patients, nor to routinely determine the complement fixing ability of detected anti-HLA antibodies. In patients who are sensitised or deemed high immunological risk, a timetable of post-transplant antibody sampling should be agreed within the transplant centre. This is in line with the recommendations set out in both the BSHI/BTS and the Transplantation Society Guidelines [22, 204].

Identifying a process likely to lead to a decline in long term transplant function will be worrying for patients/their carers. It is important to involve patients in treatment decisions including need for biopsy, alterations to the immunosuppressive regime. This may also include plans for return to dialysis or re-transplantation.

#### Guideline 5.6 – KTR: treatment of chronic allograft injury

We suggest that chronic allograft injury should be treated:

- By withdrawal of calcineurin inhibitors (CNIs) if there is either histological evidence of CNI toxicity or non-specific interstitial fibrosis and tubular atrophy (2C)
- By intensification of immunosuppression if there is evidence of ongoing immune injury (cellular rejection and/or humoral rejection) (2C)

In a similar fashion to other patients with CKD following similar preventative strategies and with timely referral to low clearance services (2D)

##### Rationale

There is some evidence that withdrawal of CNIs following chronic deterioration of graft function is beneficial [50, 158, 224]. The role of m-TOR-inhibitors as replacements for CNIs is uncertain but this approach should be avoided in patients with eGFR <40 mL/min/1.73 m^2^ and/or significant proteinuria (PCR >50 or ACR >35 mg/mmol) [185]. An overview of treatment options dictated by findings on allograft biopsy is shown in Table 2.

**Table 2:** Interpretation of the Banff Classification

Banff code	Descriptive term	Pathophysiology	Interpretation	Treatment
i	Interstitial inflammation	Infiltration of interstitium by mononuclear cells	Linked with cellular rejection but also viral infection	Intensification of immunosuppression –often pulsed intravenous steroids
t	Tubulitis	Infiltration of renal tubules by mononuclear cells	Linked with cellular rejection but also viral infection	Intensification of immunosuppression –often pulsed intravenous steroids
g	Glomerulitis	Margination of inflammatory leukocytes in the glomerular capillary loops	Marker of humeral rejection	Intensification of immunosuppression if not too much chronic damage
v	Arterial inflammation	Inflammation of arterial wall with infiltration of mononuclear cells	Marker of either severe cellular rejection or humeral rejection	Intensification of immunosuppression ifnot too much chronic damage
ptc	Peritubular capillaritis	Margination of inflammatory cells in the peritubular capillaries	Marker of humeral rejection	Intensification of immunosuppression if not too much chronic damage
ci	Interstitial Fibrosis	Interstitial structure replaced by fibrosis	Marker of chronic damage	Poor prognostic sign – may prompt reduction in CNI
ct	Tubular atrophy	Interstitial tubules involuted	Marker of chronic damage	Poor prognostic sign – may prompt reduction in CNI
cg	Transplant glomerulopathy	Interposition of mesangium and thickening of GBM	Associated with proteinuria and development of DSAs – End lesion of CAMR	Poor prognosis – no known treatment but intensification of immunosuppression often practiced
mm	Mesangial matrix expansion	Increase of thickness of mesangial matrix	Marker of microvascular damage to glomerulus	Usually interpreted in association with other findings
cv	Arterial fibrointimal thickening	Expansion of intima between endothelium and media	Marker of chronic damage – non-specific	Poor prognostic sign – vascular protective measures
ah	Arteriolar hyalinosis	Nodular deposition of hyaline	CNI toxicity but non-specific (e.g. HT, DM, lipids)	Reduction or withdrawal of CNI

There is no proven therapy for chronic humoral rejection and studies are ongoing in this area. Whilst to date there is limited high quality (e.g. randomised controlled trials) evidence to support this strategy, it seems logical to consider increased immunosuppression in the face of ongoing immunological damage to the kidney transplant. Employing measures used in other non-transplant patients with CKD is likely to be of benefit; for example, some studies show that anaemia is more prevalent in transplant patients and is associated with poor outcomes [227]. The BTS ‘Management of the Failing Kidney Transplant’ guideline covers this aspect of management in detail [23].

#### Guideline 5.7 – KTR: renal biopsy in chronic allograft injury

We suggest that a renal transplant biopsy is indicated:

- If there is a persistent unexplained elevation of creatinine or failure to return to baseline after an episode of biopsy proven acute rejection (BPAR) (1C)
- Every 7–10 days during delayed graft function (DGF) (2C)
- If expected renal function is not achieved within 4–8 weeks (2D)
- If sustained new onset proteinuria develops (PCR >50 mg/mmol or ACR >35 mg/mmol) (2C)

##### Audit Measures

- The percentage of KTRs with positive C4d staining on biopsy
- The percentage of KTRs with a donor specific HLA antibody at the time of biopsy

##### Rationale

Unexplained changes in serum creatinine are an important clinical marker of rejection and proteinuria is associated with poor outcome; both should be investigated for a treatable cause [5, 32, 72].

### Kidney Transplant Recipient (KTR): cardiovascular disease and lifestyle (Guidelines 6.1-6.6)

#### Guideline 6.1– KTR: hypertension

We suggest that the management of hypertension take into account that:

- Blood pressure should be recorded at each clinic visit (1C)
- Clinic blood pressure should be <140/90 mmHg in clinic (130/80 mmHg if PCR >50 or ACR >35) (2C)
- Home blood pressure recordings and 24-h ambulatory recordings may be helpful in some instances but lower BP targets should then be set (home and or ambulatory daytime measures <135/80 mmHg) (2D)
- There is no evidence that any antihypertensive agent is better than any other and effort should be focused on achieving absolute levels rather than the use of individual agents (2D)
- Inhibitors of the renin-angiotensin system may be more effective in the minimisation of proteinuria but they should be used with caution in the first 3 months post transplant (2C)
- Resistant hypertension may be due to transplant renal artery stenosis and should be investigated according to local practice (2D)

##### Audit Measures

- The proportion of patients receiving a target blood pressure of 140/90 mmHg or 130/80 mmHg in the presence of proteinuria PCR > 100 or ACR > 70)
- Proportion of patients with proteinuria assessed by dipstick and, if present, quantified at each clinic visit

##### Rationale

The goal of treatment of hypertension in KTR is to reduce the risk of the cardiovascular complications of hypertension (stroke, heart failure, myocardial infarction and arrhythmia) and to preserve or minimise the rate of long term decline in graft function. Blood pressure targets and strategies for treatment should be tailored to the individual patient taking into account proteinuria, the presence of end organ damage such as left ventricular hypertrophy, potential side effect profile of antihypertensive therapy, immunosuppression, and any other relevant lifestyle factors such as safety in pregnancy in female KTRs of childbearing age.

Hypertension is common following kidney transplantation and is associated with reduced graft and patient survival. Many patients require treatment with more than one antihypertensive agent and despite therapy many patients fail to meet treatment targets. Hypertension is a side effect of immunosuppressive therapy, in particular corticosteroids and calcineurin inhibitors, and may also be a consequence of poor graft function leading to salt and water retention. Defining the target optimal target blood pressure in KTRs is challenging and is mainly based on observations made from retrospective registry studies. When office/clinic blood pressure is felt to be unrepresentative of actually blood pressure (such as suspected ‘white coat’ hypertension, resistant hypertension), home recordings or ambulatory monitoring can be useful, but most studies demonstrate lower measures with these methods and revised targets should be set [218]. Resistant hypertension, often defined in the general population as clinic BP >160/90 mmHg on 3 or more antihypertensive agents is common in KTR and suggests that further work up is required to exclude transplant renal artery stensosis, non-compliance, or rarer causes of secondary hypertension e.g. primary aldosteronism or phaeochromocytoma. In the general population, there has been a move towards more frequent use of ambulatory blood pressure monitoring or home blood pressure measurements for the diagnosis and treatment of hypertension. This is a reasonable strategy for KTRs with the proviso that KTRs are known to be at higher risk of the complications of hypertension and there should be a low threshold for therapeutic intervention when blood pressure targets are exceeded. Where resistant hypertension is suspected, target end organ damage should be assessed using echocardiography to look for the presence of left ventricular hypertrophy.

There are no large scale trials of any single antihypertensive agent in KTRs. Treatment strategies are therefore either defined from extrapolation from other populations or from retrospective analysis of registry data or post hoc studies from clinical trials. The use of blockers of the renin-angiotensin system has been associated with improved patient and graft survival in some but not all retrospective studies, and effectively reduces proteinuria in KTRs although recent trial data suggest that this may not translate into better longer term graft outcomes [109, 154, 209]. However, this may be balanced against the risk of hyperkalaemia, more anaemia, and the drop of GFR when these agents are started. The drop in GFR may be predictable and if non progressive and <30% may reflect simply the haemodynamic effect of starting these agents rather than any more serious consequence of these agents. A greater drop in GFR is suggestive of transplant renal artery stenosis and may prompt further imaging. The use of dihydropyridine calcium antagonists may also have benefits in hypertension associated with use of CNIs. Modification of immunosuppression including switching from ciclosporin to tacrolimus, minimisation of calcineurin inhibitors, switching to CNI-free immunosuppression and withdrawal of corticosteroids may all be associated with lower blood pressure. All these strategies may be considered in the presence of resistant hypertension in the absence of any other cause, when graft function is stable and there have been no recent rejection episodes.

#### Guideline 6.2 – KTR: dyslipidaemia

We suggest that the management of dyslipidaemia take into account that:

- Fasting lipid levels should be measured on an annual basis in all renal transplant recipients (2C)
- Treatment targets should be the same as in the general population (2C)
- KTRs at increased primary or secondary CV risk receive statin therapy to reduce the risk of coronary artery disease (2C)
- The choice and dose of statin should take into account concurrent immunosuppression. High dose simvastatin (≥40 mg daily) should be avoided in conjunction with ciclosporin and/calcium channel antagonists (2D)

##### Audit Measures

- Proportion of renal transplant patients with measure of lipids
- Proportion of renal transplant patients taking statins for primary and secondary prevention of cardiovascular disease
- Proportion of transplant patients receiving other lipid lowering treatments
- Proportion of patients achieving dyslipidaemia targets

##### Rationale

Patients with CKD who have reached end stage renal disease (ESRD) requiring transplantation are at high cardiovascular risk. The leading cause of graft loss is death with a functioning graft, whilst the leading cause of death in renal transplant recipients is cardiovascular disease. Therefore, it is imperative that cardiovascular risk is lowered aggressively to optimise patient and graft outcomes. Kidney transplant recipients have a high prevalence of dyslipidaemia, including raised total, HDL and LDL cholesterol and hypertriglyceridaemia [94]. Dyslipidaemia is a consequence of immunosuppressive therapy, specifically corticosteroids, ciclosporin (more so than tacrolimus), sirolimus and everolimus [141]. Lipid lowering therapy is likely to beneficial for many renal transplant recipients. Unlike previous versions of these guidelines, we have taken account of the recent JBS3 guidelines, which suggest measurement of *non-fasting* lipid profile [14]. It seems sensible to use similar clinical practice for assessment of lipid status in KTR as in the general population. However, the JBS3 calculator is not likely to be appropriate for use in KTR and other calculators specific to KTR exist [197]. In keeping with the ethos of lifetime risk rather than an absolute LDL-cholesterol value being used as a trigger for intervention with lipid lowering therapy, we suggest that a 10% 7-year risk of a major adverse cardiac event using the risk calculator derived from the ALERT study, or a 10% 10-year risk of myocardial infarction or stroke using the JBS3 calculator (which is likely to underestimate risk in transplant patients) should be used as a trigger for consideration of lipid lowering therapy.

Performing a baseline assessment of lipid status allows concordance with therapy to be assessed and additionally allows estimation of the level of lipid reduction. Concordance with therapy is recognised to be challenging in renal transplant recipients who are usually on multiple agents often including immunosuppression, antihypertensive therapy and antimicrobial prophylaxis. Lipid assessment should be performed once immunosuppressive drug dosing is stable and the risk of acute rejection requiring corticosteroid therapy has fallen. This is likely to be least 3 months after transplantation although this will vary with individual patients. Specific targets for treatment have not been recommended as they are difficult to define and are not well defined in the general population. The choice and dose of statin is more likely to be selected on the basis of safety and the immunosuppressive regimen rather than potency in lowering LDL cholesterol.

Statins have similar effects on the secondary dyslipidaemia seen in renal transplant recipients as is demonstrated in primary dyslipidaemia in the general population. The ALERT study showed in a large scale randomised controlled trial that long-term treatment with fluvastatin (40–80 mg per day) compared with placebo non-significantly reduced the risk of coronary death or non-fatal MI in ciclosporin-treated renal transplant recipients [86, 87]. In this study, fluvastatin led to a significant 35% relative reduction in the risk of cardiac death or non-fatal MI. 18.7% patients in ALERT were diabetic at baseline and diabetes was a risk factor for cardiac death in this study [93]. However, there was not a significant reduction in cardiac events in diabetic renal transplant patients.

Statins are metabolised by the cytochrome P450 microsomal enzyme system and concurrent therapy with inhibitors of this system such as ciclosporin or tacrolimus can lead to greater statin exposure and higher risk of side effects such as rhabdomylosis. This risk appears to be greater with simvastatin and is lowest with fluvastatin or pravastatin [174, 201]. Ezetimibe appears to be safe in renal transplant recipients; although it has been reported to interfere with ciclosporin levels, recent reports suggest this is unlikely to be a major clinical problem [25, 208]. Fibrates have a high risk of side effects and are best avoided in renal transplant recipients.

#### Guideline 6.3 – KTR: diabetes mellitus

We suggest that the detection and treatment of diabetes mellitus in KTR should include the following:

- Screening for the development of post transplant diabetes mellitus (PTDM) by dipstick urinalysis and measurement of blood sugar level at each clinic visit (2C)
- Post transplant immunosuppression should take into account risk factors for the development of diabetes. (2C)
- We recommend that diagnosis of PTDM is made based on WHO criteria for diagnosis of diabetes mellitus based on fasting or random blood, serum HBA1c or oral glucose tolerance testing (1C)
- We suggest that a diagnosis of PTDM is made once patients are established on stable maintenance immunosuppression (2D)
- We suggest that post-transplant diabetes should be managed in collaboration with specialists in diabetic medicine (2D)
- All units should have a protocol for the management of post-transplant diabetes (2C)
- KTR with diabetes (either prior to transplantation or PTDM) should undergo screening for diabetic complications (retinal screening, foot care, neuropathy) in line with guidelines for non KTR patients with diabetes (2D)

##### Audit Measures

- Incidence of post transplant diabetes mellitus (PTDM) at 3 months and at annual intervals thereafter
- Proportion of patients who require insulin, and in whom remedial action is undertaken – minimisation of steroids and switching of CNIs
- The proportion of patients with PTDM enrolled in screening for extra-renal complications of PTDM

##### Rationale

Post-transplant diabetes mellitus (PTDM) is common following successful kidney transplantation and occurs in 5–20% of KTRs. PTDM impacts on patient survival, particularly by increasing the risk of cardiovascular disease. Data are limited on the exact magnitude of increase in cardiovascular risk associated with PTDM or the risk of microvascular complications of PTDM (including retinopathy, neuropathy and late graft failure due graft diabetic nephropathy). Risk factors for PTDM are well established and include increasing age, obesity, known glucose intolerance or metabolic syndrome, hepatitis C and family history. Transplant-specific risk factors for PTDM include tacrolimus use (compared to ciclosporin) and corticosteroids. Whilst a number of studies and prior guidelines have advocated switching immunosuppression specifically to minimise the risk of PTDM, this should be balanced against the need for optimal graft function and risk of acute rejection and subsequent need for further steroids, which may paradoxically increase PTDM risk. Similarly, steroid minimisation is an attractive strategy for reducing the risk of PTDM, but published evidence does not consistently support this.

Many patients will exhibit transient hyperglycaemia in the first month after transplantation due to a combination of high dose corticosteroids and CNI inhibition. Whilst this identifies a group of patients at risk of PTDM, it is not useful to over diagnose PTDM and it is reasonable to confirm the presence of PTDM once immunosuppression is stable, typically at 3 months post transplantation.

Once diagnosed, care of the KTR with PTDM should be co-ordinated in a similar fashion to patients with diabetes without transplants. Particular focus should be paid to maintenance of good glycaemic control, treatment of conventional cardiovascular risk factors and screening for the microvascular complications of PTDM. There is no current evidence for any particular hypoglycaemic in KTRs and lifestyle measures, oral hypoglycaemic agents and insulin can all be used to address glycaemic control.

#### Guideline 6.4 – KTR: ischaemic heart disease

- We suggest that KTRs receive standard treatment for ischaemic heart disease including thrombolysis, revascularisation, and secondary prevention (2C)

##### Audit Measures

- Proportion of patients with ischaemic heart disease
- Proportion of patients suffering myocardial infarction
- Proportion of patients undergoing primary revascularisation
- Proportion of patients receiving secondary prevention with a statin, anti-platelet agents and RAS blockers

##### Rationale

Coronary artery disease (CAD) is common in patients with ESRD, including transplant recipients, and is a major contributory factor to cardiovascular mortality and morbidity. The presence of coronary artery disease often influences the decision to transplant list patients with ESRD. Irrespective of the presence or severity of pre-transplant CAD, once transplanted, CAD is reported in 14–20% (previous myocardial infarction MI or CAD) [29, 38, 164]. Post transplantation myocardial infarction (MI) is common, affecting approximately 11.1% of patients by 3 years post transplantation, and much of this risk is experienced early, within the first 6 months of transplantation [118]. MI risk has been shown to be linked with modifiable factors including delayed graft function, post transplantation diabetes and graft failure, and in turn, MI predicts graft failure and death. Additionally, the ALERT trial [86] demonstrated that determinants of non-fatal myocardial infarction in RTR include total cholesterol level, prior CAD and previous acute rejection. Combined, these data suggest that whilst RTR share common risk factors with the general population for CAD and MI post transplantation, there are further graft-specific aspects to post-transplant CAD. It is known that patients with ESRD, including transplant recipients, are less likely to undergo cardiac intervention (bypass grafting, thrombolytics or per-catheter therapies), possibly because of higher complication rates, and are less likely to receive secondary prevention. There is no reason to believe that transplant recipients will benefit less than patients in the general population, many of whom have renal impairment. Patients with renal transplants should have equal access to cardiac investigations and surgery as patients without CKD.

#### Guideline 6.5 - KTR: smoking cessation

We recommend that smoking should be discouraged in transplant recipients (1A)

##### Audit Measures

- Proportion of KTRs who smoke
- Proportion of cigarette smoking KTRs who have been given formal advice or offer help with cessation

##### Rationale

Cigarette smoking is strongly associated with the reduced life expectancy, several forms of malignancy, respiratory disease and premature cardiovascular disease in the general population. Whilst the evidence is less comprehensive in KTRs, cigarette smoking has been shown to be associated with reduced patient survival, malignancy, and increased cardiovascular events [119, 164]. In the general population, various intervention strategies have been shown to be beneficial in encouraging smoking cessation (nicotine replacements - gum, patch, and inhaled, counselling, and Bupropion) [44, 92]. Maintenance of abstinence is challenging, as 10–20% of former smokers will resume smoking following previously successful cessation [52]. The long-term benefits of smoking cessation have not been proven in transplant recipients, nor are long-term studies likely to be performed. However, strategies for smoking cessation are safe and likely to produce the same benefits seen in other populations or public health studies. A local strategy should be available and record made of the advice given and available.

#### Guideline 6.6 KTR: lifestyle measures

We suggest that advice on healthy lifestyle forms a routine part of post-transplant care:

- Maintenance of a healthy diet should be encouraged (2C)
- An ideal weight should be targeted (body mass index (BMI) ≤25 kg/m^2^) (2C)
- Weight management services should be available (2C)
- We suggest that KTRs participate in physical activity at a level similar to that recommended to age and co-morbidity matched counterparts from the general population (2D)
- Alcohol consumption should be within national guidelines (2D)
- Recreational drug use should be avoided (2D)
- The use of over-the-counter medications (without discussion with clinical staff) and non-proprietary medications (e.g. herbal medicines) should be discouraged (2D)

##### Audit Measures

- Proportion of patients who are obese

##### Rationale

Transplant recipients have often been subjected to dietary restriction associated with advanced CKD, removal of which after transplantation is one of the factors contributing to weight gain, the metabolic syndrome, diabetes and their sequelae. Whilst metabolic syndrome and obesity have been associated with poorer graft outcomes, the overall impact on obesity on patient and graft outcomes is less than might be expected [150]. However, as longer follow up data emerge from the general population regarding the association between obesity and cardiovascular disease and malignancy [12] it would seem prudent for KTRs to avoid obesity. KTR should have access to dietary advice, and to weight management services if necessary. Pharmacological intervention for obesity has not been assessed in a clinical trial in KTR and may interfere with the metabolism and absorption of immunosuppressive agents [58]. Bariatric surgery is similarly unproven in this population and likely to have a higher incidence of side effects and potential interactions. Dose reduction or withdrawal of corticosteroids helps weight loss but more intensive monitoring is essential around the time of dose changes.

There are very limited data on any specific dietary interventions in KTRs. However, as premature cardiovascular disease is a leading cause of death following transplantation, we suggest that patients follow a diet, which minimises intake of saturated fat, sugar and salt.

We suggest that in addition to body weight control, dietary advice should be given to ensure adequate calcium and magnesium intake and control of serum phosphate level.

We suggest that KTRs avoid dietary intake of produce associated with an increased risk of infections such as *listeria monocytogenes*, *campylobacter jejuni,* etc., e.g. raw shellfish, unpasteurised dairy products. Grapefruit juice should be avoided due to the potential for interference in the metabolism of immunosuppressant drugs (tacrolimus, ciclosporin) by intestinal CYP34A, leading to increased drug levels.

KTRs have often been restricted in their ability to keep physically active whilst on dialysis. Maintaining a ‘healthy’ level of physical activity is likely to be beneficial and excise based interventions have been shown to have a positive impact on quality of life and aerobic capacity in KTRs [79]. Participation in sporting events is often beneficial. Due to the position or the transplant, participation in sports where a direct blow to the allograft is possible is not recommended (e.g. kickboxing).

Alcohol abuse occurs in a small proportion of KTRs though the prevalence and severity of alcohol misuse is difficult to define. Alcohol use within recommended guidelines after transplantation is likely to be safe, whilst alcohol or substance abuse are associated with an increased of premature death [127, 157]. Access to counselling, addiction services and rehabilitation should be available.

Non-steroidal anti-inflammatory drugs are widely available ‘over the counter’ and should be avoided. There are potential interactions with OTC and “herbal medications” (e.g. St. John’s Wort). Patients should be aware of the increased risk and potential sequelae of drug interactions, and encouraged to discuss any proposed changes to medication with clinical staff or an expert renal pharmacist.

### Kidney Transplant Recipient (KTR): neoplasia (Guidelines 7.1-7.3)

#### General concepts

Neoplasia is more common in KTRs due to impaired immunosurveillance. In particular, virally driven cancers are more prevalent e.g. human papilloma (HPV)-induced cervical cancer (see Table 3). The relative risk of cancer is higher in younger patients (20x relative risk of neoplasia) than older patients (2x for over 65 s) [127, 157]. KTRs with neoplasia have worse outcomes than members of the general population, probably due to increased toxicity from treatment. Preventative strategies are therefore paramount, which may involve screening and the minimisation or modification of immunosuppressive therapy. If cancer develops then part of the treatment will involve reducing and/or modifying immunosuppressive therapy. This is likely to be more beneficial in those cancers with higher relative risks in KTRs (e.g. more likely to have a clinical impact in non-melanoma skin cancer than pancreatic cancer). Emerging evidence supports the notion that low dose immunosuppression and the use of m-TORi may reduce the incidence and recurrence of some cancers [31, 148, 228].

**Table 3:** Association of risk of common malignancies in KTRs

Relative risk	KTR Common cancers
High RR >5	Kaposi’s sarcoma
	Eye
	Lymphoma
	Kidney
	Non-melanoma skin
	Lip
	Thyroid
Medium RR 1-5	Melanoma
	Cervix
	Vulvovaginal
	Bladder
	Colon
	Lung
	Stomach
	Oesophagus
	Oropharynx and Larynx
	Myeloma
	Anus
	Leukaemia
	Hepatobiliary
No increase	Breast
	Prostate
	Ovary
	Uterus
	Pancreas
	Brain
	Testis

#### Guideline 7.1 – KTR: screening for cancer

We suggest that the organisation of screening for neoplasia in KTRs take into account:

- Screening should be similar to the general population for cervical, breast, colon and prostate cancer (2C)
- Screening is not recommended for renal cell carcinoma (2C)
- Patient education pre and post transplantation (1C)
- Patients should be aware of malignancy risk and encouraged to report symptoms which may represent *de novo* malignancy (e.g. breast or testicular lumps) (2D)
- Skin surveillance by a healthcare professional at least biannually up to 5 years post-transplant and annually from 5 years post-transplant (2C)
- Patients with cirrhosis should undergo an annual hepatic ultrasound and determination of serum alpha feto-protein (2C)

##### Audit Measure

- Proportion of patients having screening procedures for neoplasia at the annual review clinic

##### Rationale

The merits of any screening programme must balance the individual’s risk of developing the disease, their prognosis if detected, and the risk of harm from screening. Screening should be individualised and reflect co-morbidities and other competing risks (e.g. vascular disease). Some authors have advocated more frequent screening (e.g. annual cervical screening) but there is little evidence to support these assertions. Thus screening should follow the pattern in the general population for most common cancer [159]. The national cancer screening protocols are described on the NHS Cancer Screening Programme (NHSCSP) website [148]. Patient education regarding neoplasia should be undertaken pre-transplant, with reinforcement post-transplant. Education should include breast, testicular and skin self- examination. Information about risk factors for the development of NSMC should also be included [74].

That all KTRs should undergo skin surveillance is not in doubt but the frequency of survey to optimally balance early detection and treatment with finite resource is unclear [64]. The incidence of non-melanoma skin cancers increases with duration post-transplant and skin surveillance should therefore be more frequent [74, 170]. Encouragement of self-examination and education should be reinforced in the initial years following transplant, supplemented with skin surveillance by a trained healthcare professional at least biannually [200]. From 5 years post-transplant, skin surveillance should be undertaken annually to reflect the increased likelihood of developing NSMC [170] Smoking cessation should be encouraged in all KTRs. A formal protocol for the management of smoking cessation should be available in each transplant centre (see Guideline 6.5 – KTR: smoking cessation)

#### Guideline 7.2 – KTR: Non-Melanoma Skin Cancer (NMSC)

We recommend that KTRs should be educated about the adverse effects of solar exposure (1C)

#### Guideline 7.3 – KTR: Non-Melanoma Skin Cancer (NMSC)

We suggest that KTRs that an individualised assessment of hazard should be made according to risk factors (2C)

#### Guideline 7.4 – KTR: Non-Melanoma Skin Cancer (NMSC)

We recommend that patients should be encouraged to cover their skin in direct sunlight and to use total sunblock (Sun Protection Factor ≥ 50) (1D)

#### Guideline 7.5 – KTR: Non-Melanoma Skin Cancer (NMSC)

We suggest that self-examination should be encouraged with guidance provided. This should be supplemented by at least biannual review by a trained healthcare professional up to 5 years post-transplant and annual review from 5 years (2C)

#### Guideline 7.6 – KTR: Non-Melanoma Skin Cancer (NMSC)

We suggest that the prescription of acitretin as chemoprophylaxis be considered in those with ≥2 previous NMSC if there are no contraindications (2B)

##### Rationale

Certain patient groups are at higher risk of non-melanoma skin cancer, particularly the fair-skinned living in a sunny climate. Other risk factors include occupation, behaviour, previous skin cancer, childhood solar exposure and family history. It is sensible to minimise exposure and use sun block. Acitretin (0.2–0.4 mg/kg/day) may reduce total NSMCs in those who have had ≥2 previous NSMCs and thus use should be considered in those with previous skin cancer [97]. There is some evidence that sirolimus reduces the incidence of second tumours but at the expense of increased side effects and possibly adverse effects on graft function [101, 183, 199]. Recent studies suggest that m-TORi may be associated with fewer NMSC, particularly cutaneous Kaposi sarcoma and recent data suggest that switching KTRs to sirolimus is associated with reduction of secondary NMSC [57, 183]. The role of HPV vaccination in KTRs is unclear, but it is an inactivated vaccine that can be administered safely either before or after transplantation [111].

#### Guideline 7.7 – KTR: immunosuppression in cancers

We suggest that the overall level of immunosuppression should be reduced if neoplasia develops (2C)

#### Guideline 7.8 – KTR: immunosuppression in cancers

We suggest that m-TORis are considered as alternative immunosuppressive agents in KTRs who develop *de novo* maligancy (2C)

#### Guideline 7.9 – KTR: immunosuppression in Kaposi’s sarcoma

We suggest that m-TORis has specific anti-tumour effects in Kaposi’s sarcoma and switching to this medication should be considered (2C)

##### Rationale

There is no evidence that any particular immunosuppressant agent is linked to a particular cancer other than the association of TDAs and PTLD [106, 148]. It is generally agreed that the level of immunosuppression should be decreased when cancer occurs. This decision should be individualised according to the stage of the cancer at diagnosis, the likely impact of a reduction in immunosuppression, the availability of treatment for the tumour, and potential drug interactions between the chemotherapy and immunosuppressive agents. In general, the effect of reducing the overall level of immunosuppression is more likely to be beneficial where the relative risk of the tumour in KTRs is higher. m-TORis are indicated in the treatment of Kaposi sarcoma in addition to reduced levels of overall immunosuppression [200].

## Kidney Transplant Recipient (KTR): infection complications (Guidelines 8.1–8.7)

### Guideline 8.1 – KTR: vaccination

#### Guideline 8.1.1 – KTR: vaccination

We recommend that KTRs:

- Should be vaccinated with inactivated viruses as per the normal population (1D)
- Should receive annual influenza vaccination unless contraindicated (1C)

#### Guideline 8.1.2 – KTR: vaccination

We suggest that KTRs:

- Should have HBsAb levels rechecked annually and consider revaccination if antibody titres fall below 10mIU/ml (2D)
- Should not receive live attenuated vaccines (2C)
- Should receive pneumococcal vaccine and a booster every 5 years (2D)

##### Rationale

Vaccination for KTRs and their household members should ideally be completed prior to transplantation. A minimum of 4 weeks is recommended between vaccination with live attenuated vaccines and transplantation. After transplantation, there is no evidence to link vaccination with rejection. After transplantation it is safe to administer inactivated vaccines but live attenuated vaccines should be avoided (see Table 4 below) as small studies have demonstrated concerns regarding their safety and efficacy in KTRs. Vaccination should probably be carried out at least 3 months and preferably 6 months after transplantation when the immunosuppression has been reduced. Consideration should be given to vaccination of close household contacts where appropriate e.g. Varicella vaccination for children of VZV seronegative KTRs.

**Table 4:** Commonly used inactive and live attenuated vaccines

Inactive Vaccine	Live Attenuated Vaccine
Inactivated Influenza	Varicella Zoster
Hepatitis A	Mumps
Hepatitis B	Rubella
Inactivated Polio	Measles
Diptheria	BCG
Tetanus	Smallpox
Meningococcal	Yellow Fever
Pneumococcal	Oral Salmonella
Human Papilloma Virus	Oral Polio
Rabies	
Anthrax	
Intramuscular Salmonella	
Japanese encephalitis	
Inactivated intravenous cholera vaccine	

The HPV vaccine has been assessed in a small study of transplant recipients. Although the vaccine was safe and tolerated, immunogenicity was suboptimal [113]. There is a strong link between HPV and anogenital and non-melanoma skin cancer and, given the tolerability of these vaccines, some authors have recommended vaccination for all female KTRs aged between 9 and 26 [35]. Malaria prophylaxis should consist of chloroquine in sensitive areas, but it may increase levels of ciclosporin. Prophylaxis should therefore start 2 weeks prior to departure to permit monitoring of drug levels. In areas of chloroquine-resistance three options can be used: atovaquone and proguanil; mefloquine; or doxycycline. The choice of agent will be dictated by local preference and side effect profile but drugs should be started a few weeks prior to departure to allow for checks of renal and hepatic function, full blood count and immunosuppression levels. More extensive guidance is available for KTRs who are travelling overseas [77].

### Guideline 8.2 – KTR: cytomegalovirus disease

#### Guideline 8.2.1 – KTR: prophylaxis and treatment of CMV disease

We recommend:

- Prophylaxis should be continued for 3–6 months, until immunosuppression has been reduced to long-term maintenance level (1B)
- Treatment should be administered for 6 weeks after treatment with a TDA (1C)

#### Guideline 8.2.2 – KTR: prophylaxis and treatment of CMV disease

We suggest:

- All transplant units should have the ability to measure CMV serological status and the detection and quantification of viral load (2D)
- Donor and recipient CMV sero-positivity should be recorded at the time of transplantation (2D)
- A written protocolised strategy based either on prophylaxis, or pre-emptive therapy, or both should be implemented (2D)
- For the treatment of mild and moderate CMV disease, oral valganciclovir and intravenous ganciclovir are of equivalent efficacy (2C)
- Treatment of life-threatening CMV disease should be initiated with intravenous ganciclovir (2D)
- Treatment duration should be determined by monitoring viral load (2C)

##### Audit Measure

- Incidence of CMV disease.

##### Rationale

CMV infection is the most common serious viral infection affecting renal transplant recipients [89, 110]. It occurs most commonly in CMV naïve recipients of a kidney from a CMV positive donor. However, seropositive transplant recipients may be affected by reactivation of CMV infection and by primary infection with a new genotype. CMV infection is associated with more intensive immunosuppression, treatment of acute rejection episodes and the use of TDAs. For CMV disease there is clear evidence that prophylaxis reduces the severity, delays the onset and prevents CMV infection in CMV negative recipients of CMV positive kidneys [85, 125]. CMV infection is also associated with concomitant infection by other herpes viruses, the significance of which is uncertain but prevention of which may be an added benefit of prophylaxis over pre-emptive strategies [90]. CMV prophylaxis is therefore recommended in CMV negative KTRs from a CMV positive donor (D+/R-), and for seropositive KTRs (D-/R+ or D+/R+) exposed to more intensive immunosuppression, in particular TDAs.

In CMV negative recipients of a CMV positive kidney the use of oral antiviral therapy – specifically valaciclovir, aciclovir, ganciclovir, or valganciclovir – is proven to delay the onset of CMV disease, to prevent CMV disease in a proportion of patients, and to limit the severity of disease. It is important to recognise that a proportion of patients will develop CMV disease after stopping prophylaxis and will require clinical and virological monitoring for at least 3 months following the stopping of prophylactic therapy [90]. At present, evidence exists for the use of valaciclovir and valganciclovir [91, 212]. In clinical practice, valaciclovir is not widely available or used, and valganciclovir is the agent of choice. The dose of valganciclovir should be adjusted according to renal transplant function. Most commonly, and based on the available evidence, antiviral prophylaxis is usually continued for 90–180 days following transplantation (depending on D and R status). The rationale is that reduction of immunosuppressive therapy over this period will allow the immune system to combat viral replication once prophylactic therapy is withdrawn [9, 126].

Treatment of CMV disease is equally effective with either oral valganciclovir or intravenous ganciclovir [77, 89]. However, this study excluded patients with life-threatening CMV infection and it is probably advisable to initiate treatment with intravenous therapy in such circumstances [110].

### Guideline 8.3 – KTR: Epstein Barr virus infection

#### Guideline 8.3.1 – KTR: EBV infection

We recommend that immunosuppression should be reduced or stopped following the development of PTLD (1C)

#### Guideline 8.3.2 – KTR: EBV infection

We suggest:

- Both donor and recipient should have their EBV serology recorded at the time of transplantation (2D)
- All high risk (D+/R-) patients (including adults) should have EBV viral load measured immediately after transplantation, monthly for 6 months, and 3 monthly to 1 year (2C)
- EBV viral load should be monitored after the treatment of rejection (2C)
- Total immunosuppression should be reduced when EBV titres rise significantly (2C)

##### Audit Measures

- Rates of EBV infection and PTLD amongst KTRs
- Completeness of records for EBV donor and recipient serology

##### Rationale

After transplantation, primary EBV infection may present with a broad spectrum of disorders ranging from asymptomatic infection to high grade non-Hodgkin lymphoma (PTLD). EBV seronegative KTRs, undergoing seroconversion post transplant, are up to 50 times more likely to develop PTLD compared to their seropositive counterparts. The EBV genome is found in more than 90% of PTLD occurring during the first year after transplantation [3]. Vigilance is therefore essential, especially since EBV viraemia usually precedes the development of PTLD by 4–16 weeks [178]. However, assays for EBV viral load can often be positive in asymptomatic patients (false positives) and so clinical correlation and attention to changes in viral load are essential. Risk factors for early PTLD include primary EBV infection, young donor age, CMV infection, and induction with TDAs. The use of antiviral agents (e.g. valaciclovir or valganciclovir) or immunoglobulins in response to rising viral loads is unproven and cannot be recommended. Since the immune response to EBV infected tissue is thought to depend on EBV-specific T cell responses it is logical to reduce immunosuppressive treatment in the face of clinical EBV infections and PTLD.

### Guideline 8.4 – KTR: Varicella Zoster Virus infection

#### Guideline 8.4.1 – KTR: VZV infection

We recommend:

- Primary infection (chickenpox) should be treated with intravenous aciclovir or oral valaciclovir until the lesions scab over (1C)
- Uncomplicated shingles should be treated with oral acyclovir or valaciclovir until the lesions scab over (1D)
- Disseminated (>2 dermatomes), ocular or invasive shingles should be treated with intravenous aciclovir until the lesions scab over, together with a reduction in immunosuppression (1B)
- Varicella-susceptible KTRs (i.e. VZV IgG negative) with primary exposure to VZV should receive intravenous immunoglobulins, ideally within 96 h, but up to a maximum of 10 days following exposure. If unavailable or after 10 days, oral aciclovir should be administered for seven days (1D)

#### Guideline 8.4.2 – KTR: VZV infection

We suggest:

- Patients on the waiting list who are VZV IgG seronegative should be vaccinated prior to transplantation (2D)
- Immunosuppression should be reduced during primary infection (2D)

##### Audit Measures

- Annual rates of primary VZV and shingles infection
- Completeness of records for VZV recipient serology in KTRs

##### Rationale

Acquired by 90% of the population before adulthood, primary infection with VZV causes chickenpox. Thereafter the virus remains latent in the cranial nerve and dorsal root ganglia. Secondary reactivation results in shingles and typical dermatomal blistering skin lesions. Primary infection can be acquired by direct skin contact and by airborne droplet transmission [78, 160]. Primary disease in KTRs can be devastating with severe skin lesions, widespread visceral involvement and disseminated intravascular coagulation. In immune naïve and immunosuppressed individuals, treatment with pooled varicella zoster immunoglobulin has been shown to prevent or ameliorate VZV infection. It seems sensible to vaccinate VZV naïve patients on the waiting list since the vaccine has been shown to be safe [160]. As a live attenuated vaccine, VZV vaccine should not be administered post transplant.

### Guideline 8.5 – KTR: Herpes Simplex Virus infection

#### Guideline 8.5.1 – KTR: HSV infection

We recommend:

- Superficial HSV (1 or 2) infection should be treated with appropriate oral agents until the lesions have resolved (1D)
- Systemic HSV infections should be treated with intravenous aciclovir and a reduction in immunosuppression until a response occurs and oral medication continued for at least 14 days (1C)

#### Guideline 8.5.2 – KTR: HSV infection

We suggest that KTRs suffering frequent recurrent HSV infection should consider oral prophylaxis (2D)

##### Audit Measure

- Rates and outcomes of HSV infections

##### Rationale

There is an increased potential for superficial HSV infections to become disseminated or invasive in KTRs. Reactivation most commonly occurs in the first few weeks after transplantation and complicated disease can become life threatening involving multiple organs including the liver, lung and central nervous system. Since treatment is safe and effective, it seems sensible to treat early infections [172]. Due to their gravity, complicated infections should be treated with intravenous therapy and reduction in immunosuppression.

### Guideline 8.6 – KTR: BK nephropathy

#### Guideline 8.6.1 – KTR: BK nephropathy

We recommend that confirmed BK nephropathy should be treated by reduction in immunosuppression (1D)

#### Guideline 8.6.2 – KTR: BK nephropathy

We suggest:

- Screening should also be carried out when renal function deteriorates in an unexplained fashion (2D)
- KTRs should be screened for BKV viral load or by performing urine microscopy for decoy cells or by PCR on urine or serum (2C)
- Suspected BK nephropathy should be confirmed by renal biopsy, which should be stained for SV40. Two cores containing medullary tissue should ideally be examined (2D)
- Immunosuppression should be reduced when the serum BKV load exceeds 10^4^ copies/ml (2C)
- There is no established specific treatment for BK nephropathy (2D)
- Re-transplantation can safely be considered in patients who have BK nephropathy diagnosed in an earlier graft (2C)

##### Audit Measures

- Rates of BK viral infection in screening tests
- Rates and outcomes of BK nephropathy

##### Rationale

The human polyoma BK virus is linked to two major clinical syndromes in KTRs, namely BK nephropathy (BKN) and transplant ureteric stenosis [15, 43, 169]. BKN occurs in up to 10% of KTRs and is responsible for a significant number (15–50%) of allograft losses. 90% of young adults have serological evidence of prior infection and the DNA virus remains latent in the uroepithelium. The virus becomes active and replicates under the influence of immunosuppression. BK virus is cytolytic and so epithelial cells are shed in the urine as decoy cells and free virus can be detected in the urine. With increased viral replication, BKV spills into the blood and can be detected as BK viraemia by PCR. Approximately half of patients with high level viruria (>10^7^ copies/mL) will develop significant BK viraemia (10^4^ copies/mL) and half of these will develop histological BKN. Risk factors for BKV include not only both donor and recipient characteristics but also high immunosuppressive burden and intensification of immunosuppression. Definitive diagnosis requires demonstration of the virus in renal tissue, usually by staining with the antibody for large T antigen of SV40. Since the infection can be focal and preferentially affects the renal medulla, two cores including medulla should be examined [48, 83]. The mainstay of treatment is reduction of immunosuppression but there is no evidence that reducing any particular immunosuppressive agent is particularly beneficial [75, 82]. Common approaches include stopping anti-proliferative agents or reducing CNI levels in the face of rising viral replication [95]. Specific agents such as intravenous immunoglobulin, quinolones, cidofovir and leflunomide have been shown to have anti-viral activity but there is no definitive evidence to show that they offer any advantage over simply reducing the total immunosuppressive burden [130, 155, 171]. Prospective trials are urgently required to explore this question. If a first graft is lost due to BKN there is no evidence that this will adversely affect the outcome of subsequent grafts and no special precautions are necessary (e.g. allograft nephrectomy) prior to re-listing [46].

#### Guideline 8.7 – KTR: pneumocystis jirovecii infection- treatment and prophylaxis

We suggest:

- All patients with confirmation (microscopy or PCR) of Pneumocystis jirovecii in respiratory secretions should be treated for 14 to 21 days with co-trimoxazole orally or intravenously (15–20 mg/kg in three or four divided doses) (2B)
- Patients with contraindications to treatment with co-trimoxazole should receive pentamidine (4 mg/kg/day intravenously) (2B)
- Adjunctive glucocorticoid therapy may be considered in patients with severe disease (2D)
- All patients should receive 3–6 months of treatment with co-trimoxazole 480 mg daily for Pneumocystis jirovecii prophylaxis following renal transplantation (1B)

##### Rationale

Diagnosis of Pneumocystis pneumonia (PCP) requires confirmation by microscopy or PCR from respiratory secretions (induced sputum after inhaled saline or bronchoalveolar lavage). In non-HIV infected individuals, co-trimoxazole has been shown to be effective. Co-trimoxazole has excellent bioavailability and enteral administration is preferred, although intravenous therapy may be necessary in acutely unwell patients. In patients with contraindications to co-trimoxazole, intravenous or aerosolised pentamidine is a suitable alternative although it is associated with a higher rate of side effects. Alternative agents against pneumocystis jirovecii include dapsone or atovaquone. The duration of therapy should be between 14 and 21 days. The use of additional glucocorticoid therapy is not associated with improved survival in non-HIV patients, although treatment with prednisolone has been associated with quicker recovery from severe PCP infection in one small observational study [156]. As a result, PCP prophylaxis with co-trimoxazole is recommended and has shown to be beneficial in meta-analysis of 12 randomised studies in 1245 immunocompromised individuals [67].

#### Guideline 8.8 – KTR: post-transplant infection prophylaxis

- All patients should receive 3–6 months of treatment with co-trimoxazole 480 mg daily (1B)
- Oral antifungal prophylaxis should be administered for 1 week month after transplantation (2C)
- In selected patients, prophylaxis against mycobacterium tuberculosis with daily isoniazid (supplemented with pyridoxine) should be instituted for 6 months after transplantation (2C)

##### Rationale

In addition to benefit for PCP, there is good evidence that co-trimoxazole provides effective prophylaxis against urinary tract infections after renal transplantation [42, 63]. Alternatives include cephalosporins and fluoroquinolones. Alternative agents against pneumocystis jirovecii include dapsone, atovaquone or aerosolized pentamidine.

Candida infection is common after renal transplantation and can cause considerable morbidity. It is usually acquired from colonisation of the oral mucosa and so topical oral preparations offer a simple form of prevention without the potential toxicity of systemic preparations.

Tuberculosis in KTRs is usually due to reactivation of quiescent disease under the influence of immunosuppression. In other immunosuppressed populations treatment of latent tuberculosis prevents progression to clinically active tuberculosis. A recent Cochrane Database review recommended prophylaxis with isoniazid (for up to 1 year) in selected recipients at high risk of tuberculosis (based on prior infection or exposure, or patients whose country of origin has a high incidence of tuberculosis infection) with careful monitoring of liver function, especially in patients who are hepatitis B and C positive [2, 42].

#### Guideline 8.9 – KTR: Hepatitis E Virus

We recommend that Hepatitis E Virus (HEV)-screened blood components should be given to all KTR (1C)

##### Rationale

There has been an increase in the number of reports of cases of hepatitis E virus (HEV) in the United Kingdom. The infection is usually mild and self- limiting in the immunocompetent but there is increasing evidence that HEV infection in patients receiving immunosuppression may lead to persistent infection, which may lead to chronic hepatitis and cirrhosis. HEV may be transmitted through the use of blood and blood products, through transplantation and through diet (especially inadequately cooked pork and pork products such as sausages and offal). Liver tests may be normal or show mild hepatitis. The favoured diagnostic test is by HEV polymerase chain reaction. Liver biopsy may show histology. Conventional management of HEV infection is review immunosuppression and reduce or minimise immunosuppressive burden where possible. If there is no serological evidence e clearance within 3 months, consider a 3-months course of Ribavarin (note this use is off licence) [181, 182].

### Kidney Transplant Recipient (KTR): bone and joint disease (Guidelines 9.1-8.7)

#### Guideline 9.1 KTR: osteoporosis

We suggest:

- KTRs suffering from osteoporosis or at high potential risk should be considered for steroid-avoiding immunosuppression (2D)
- KTRs on longterm steroids or at high risk for osteoporosis should undergo DEXA scanning if eGFR >30 ml/min/1.73 m^2^ (2D)
- treatment should be according the RCP guidelines for steroid-induced osteoporosis (2D)

##### Audit Measures

- Prevalence of KTRs on corticosteroids
- Frequency of bisphosponate usage amongst KTRs
- Incidence of fractures amongst KTRs

##### Rationale

All KTRs have a complex bone disorder whereby the effects of immunosuppression are superimposed on an underlying Chronic Kidney Disease Mineral and Bone Disorder (CKD-MBD). Any guidance should be used in conjunction with existing guidelines for CKD-MBD [104]. The risk of fractures after renal transplantation is high but there is no accurate way to predict fracture risk. Clinical tools have not been validated in KTRs. Bone Mineral Density may not reflect the future risk of fracture in KTRs, particularly in those with eGFR <30 mL/min/1.73 m^2^ [201]. In addition bisphosphonates are contraindicated in subjects with eGFR <30 mL/min/1.73 m^2^. There is evidence that treatment with calcium and vitamin D derivatives attenuates post-transplant bone loss and maintains bone mineral density without excess hypercalcaemia [96, 175, 207]. Corticosteroids seem to be the principal determinant of bone turnover and bone volume so it seems logical to target interventions towards reduction or withdrawal of these drugs [175]. There are numerous guidelines for corticosteroid induced osteoporosis including those of the Royal College of Physicians and it seems reasonable to follow them [40, 175]. Newer agents such as denosumab, a monoclonal antibody, which inhibits RANK ligand, seem to be safe in renal impairment with the caveat that serum calcium needs to be closely monitored, as the risk of hypocalcaemia is increased [13]. One randomised control trial of denosumab in the KTR showed some benefit on preservation of bone mineral density, but at the expense of greater numbers of urinary tract infection [17].

#### Guideline 9.2 KTR: tertiary hyperparathyroidism

We suggest:

- Severe hyperparathyroidism should be treated prior to transplantation (2D)
- Cinacalcet can be used in KTRs (2C)
- We suggest treatment should be the same as for other patients with CKD (2D)

##### Audit Measures

- Incidence of hyperparathyroidism
- Incidence of parathyroidectomy
- Usage of cinacalcet

##### Rationale

Post-transplant hyperparathyroidism is a complex entity that may represent a true high bone turnover state but also low bone turnover [18]. Dietary intake of phosphate, longstanding phosphate retention whilst on dialysis treatment, followed by sometimes profound urinary phosphate losses in the early post transplant period all contribute to the complexity of managing this condition. In the latter case the suppression of parathyroid hormone (PTH) secretion may lead to adynamic bone disease and the only certain way to distinguish between the two types of mineral and bone disorder (MBD-CKD) is by bone biopsy. There are contradictory data on the effect of parathyroidectomy post transplantation but it seems sensible to treat severe hyperparathyroidism prior to transplantation [59, 100]. In KTRs, cinacalcet may be used; it successfully corrects hypercalcaemia and elevated PTH level, seems to improve bone mineral density, and may also have an antihypertensive effect [11, 198, 233]. Caution should be exercised with high doses [187]. In hyperparathyroidism refractory to pharmacological agents, parathyroidectomy also provides a benefit but should only be pursued when potential risks of such a procedure are considered.

### Guideline 9.3 gout

#### Guideline 9.3.1 treatment of gout

We recommend that neither allopurinol nor febuxostat should not be administered with azathioprine (1B)

#### Guideline 9.3.2 –KTR: treatment of gout

We suggest:

- Hyperuricaemia should be treated when associated with gout, tophi or uric acid stones (2D)
- Non steroidal anti-inflammatory drugs (NSAIDs) should be avoided in KTRs (2D)
- Episodes of gout may be treated with brief courses of oral prednisolone (2D)
- Colchicine is an effective treatment for gout in KTRs (2D)

##### Audit Measure

- Frequency of gout and hyperuricaemia amongst KTRs

##### Rationale

Gout is common after transplantation and may cause significant morbidity. Hyperuricaemia increases the risk of gout and may also be linked with increased rates of cardiovascular disease [1]. In CKD, febuoxstat may have superior urate lowering effects compared with allopurinol [188]. This observation is also borne out in the transplant population where febuxostat appears to effectively lower urate levels without adversely affecting renal transplant function, although long term outcome data are lacking [195, 205]. Important drug interactions alter the strategy for managing gout in KTRs. In particular, use of concomitant use of such agents with azathioprine can precipitate potentially fatal blood dyscrasias. CNIs are associated with higher uric acid levels and may contribute to the development of gout.

#### Guideline 9.4 –KTR: calcineurin inhibitor bone pain

We suggest:

- Reducing or withdrawing CNIs should be considered in KTRs with intractable bone pain (2D)
- Dihydropyridine calcium antagonists also may be beneficial (2D)

##### Rationale

It has become increasingly recognised that CNIs may cause bone pain, which preferentially affects bones in the lower legs [37, 69]. Bone marrow oedema can be demonstrated on magnetic resonance imaging (MRI) scanning and treatment involves reducing CNI levels and the use of dihydropyridine calcium antagonists [55].

### Kidney Transplant Recipient (KTR): haematological complications (Guidelines 10.1–10.3)

#### Guideline 10.1 –KTR: anaemia

We suggest that chronic anaemia should be managed in the same way as other patient with CKD (2D)

##### Audit Measure

- Prevalence of anaemia amongst KTRs

##### Rationale

Anaemia is common in the KTR population, with reports suggesting a prevalence of 20–30% of KTRs, and may be associated with poor outcomes [73, 227]. Its aetiology is multifactorial and may include relative erythropoietin deficiency related to suboptimal graft function, haematinic deficiency, post-transplant infection, and adjunctive antimicrobial therapy (valganciclovir, co-trimoxazole). It may be exacerbated by immunosuppressant therapy, especially antiproliferative agents, and these may be tapered or stopped to improve haemoglobin levels. Management should be similar to other patients with CKD [49]. There is one small randomized controlled trial suggesting benefit with haemoglobin correction using an erythropoeisis-stimulating agent [34]. However, this is in contrast to larger similar studies in the non-transplant CKD patients where haemoglobin correction to similar levels was associated with adverse outcomes such as increased stroke risk [162].

#### Guideline 10.2 – KTR: − polycythaemia

We recommend that initial treatment should be with angiotensin converting enzyme inhibitors (ACEIs) or with angiotensin receptor blockers (ARBs) (1C)

#### Guideline 10.3 – KTR: − polycythaemia

We suggest:

- Haemoglobin levels should be monitored at every clinic visit (2D)
- Treatment should be initiated if the haematocrit or packed cell volume exceeds 52% in men and 49% in women (2D)
- Venesection may be used in refractory cases (2D)

##### Audit Measure

- Prevalence of post transplant erythrocytosis amongst KTRs

##### Rationale

Post-transplant polycythaemia (erythrocytosis) is common after renal transplantation and may be associated with significant morbidity and mortality [103, 217]. The overall effect on long term outcome is less clear, although it is likely that it increases risk of thrombotic events [232]. Studies have shown that ACEIs and ARBs are associated with a drop in haematocrit in KTRs [69].

### Kidney Transplant Recipient (KTR): reproductive issues (Guidelines 11.1-11.5)

#### Guideline 11.1 – KTR: conception and contraception (female)

We recommend that MPA-containing immunosuppressant drugs should be stopped prior to conception and replaced appropriately (1A)

#### Guideline 11.2 – KTR: conception and contraception (female)

We suggest:

- KTRs should wait for 1 year after transplant and have stable function before attempting conception (2C)
- Counselling regarding fertility and reproduction should be offered to female KTRs and their partners either prior to transplantation or soon afterwards (2D)
- m-TORi should be stopped prior to conception and replaced as appropriate (2D)
- Pregnancy should be managed jointly with an Obstetrics department with experience of care of KTRs (2D)
- We suggest that KTRs receive aspirin 75 mg daily to reduce the risk of pre-eclampsia from 12 weeks gestation until birth of the baby unless there are contraindications (2C)
- The risks and benefits of breastfeeding should be discussed (2D)
- Contraception advice should be similar to the general population (2D)

#### Guideline 11.3 – KTR: conception and contraception (male)

We recommend:

- Male KTRs are advised that MPA containing compounds have theoretical teratogenic potential in men taking these agents (1D)
- KTRs should be advised that m-TORi reduce the male sperm count and counselled accordingly (1C)

#### Guideline 11.4 – KTR: conception and contraception (male)

We suggest:

- All immunosuppressive drugs other than m-TORi can be used in male KTRs. Advice for MPA is as Guideline 11.3 (2D)
- The decision to continue MPA containing compounds in a male KTR wishing to conceive should balance a the risk of theoretical teratogenicity against the risk of rejection on changing from MPA to azathioprine (2D)
- Men on m-TORi who wish to conceive should discontinue these agents prior to conception and replace them as appropriate (2D)
- Men who wish to maintain fertility should avoid m-TORi or bank sperm prior to starting these drugs (2D)

##### Audit Measure

- Pregnancy rates and outcomes should be monitored

##### Rationale

Female fertility returns rapidly after successful renal transplantation and KTRs and their partners need to be counselled about potential pregnancy [139]. Pregnancies in KTRs should be deemed above average risk with increased rates of maternal hypertension, pre-eclampsia, prematurity, low birth weight and caesarean section [19, 41]. The risk of pregnancy to allograft function is probably small, particularly with good baseline function [19]. Immunosuppressive drugs can all have effects on the foetus and appropriate caution should be exercised. Sirolimus and MPA compounds are teratogenic and should be avoided in females [7, 41]. Recent advice from the MHRA also advises that MPA compounds should be avoided in male KTRs prior to conception as there is a theoretical risk of teratogenicity associated with males fathering children whilst receiving these drugs [194]. This advice does not appear to be confirmed in registry studies detailing outcomes in pregnancies fathered by KTRs [194]. Therefore, in men, any theoretical risk of foetal abnormality associated with use of MPA compounds, should be balanced against the risk of rejection or decline in graft function associated with changing immunosuppression. Further information on this issue has been generated by the Renal Association’s Pregnancy and Chronic Kidney Disease Rare Disease Group and is available on the website RareRenal.org: http://bit.ly/Mycophenolate_recs.

Alternative immunosuppression should therefore be considered prior to conception. All immunosuppressants are excreted in breast milk, albeit in tiny quantities, and are usually contraindicated. However, toxicity has not been reported after breastfeeding with ciclosporin, prednisolone, azathioprine and tacrolimus [101, 232]. Aspirin is now widely recommended for the prevention of pre-eclampsia in high-risk populations, and whilst there are no specific data to support its use in KTRs, it seems reasonable to follow this approach, adopted from other guidelines [20, 143]. Outcomes of pregnancies fathered by male KTRs are similar to the general population although reporting bias is difficult to account for [51, 220].

There are very little data surrounding the use of female contraception in KTRs and so it seems sensible to extrapolate from the general population with similar cautions and contraindications [61]. A number of hypothetical risks associated with specific forms of contraception have not been confirmed in observational studies, though the data quality is poor [8].

Whilst ESRD is a state of reduced fertility, the rates of female subfertility and infertility in KTRs are largely unknown. There are a small number of case reports of successful outcomes following in vitro fertilisation in KTRs, albeit with similar complications as seen in KTRs with conventional pregnancy [159]. Further research is needed in this area. Sirolimus and presumably other m-TORi are associated with oligospermia, which appears to be reversible on cessation of treatment [8, 151, 159].

#### Guideline 11.5 – KTR: sexual function

We suggest:

- Men should be counselled about the possible risks of impotence following transplantation surgery that involves the internal iliac artery (2D)
- Specific enquiry should be made regarding sexual dysfunction, preferably at an annual review clinic (2D)
- Care pathways for dealing with sexual dysfunction should be established (2D)
- Sildenafil is safe and effective in male KTRs not taking nitrates (2D)

##### Audit Measures

- Prevalence of sexual dysfunction in the transplant clinic

##### Rationale

Sexual dysfunction is very common in both men and women with advanced CKD and manifests with decreased libido and erectile dysfunction. These problems are often improved after successful renal transplantation but remain common [62, 131, 168]. Sildenafil is safe and may be effective for erectile dysfunction in KTRs [190].

## Lay Summary

These guidelines cover the care of patients from the period following kidney transplantation until the transplant is no longer working or the patient dies. During the early phase prevention of acute rejection and infection are the priority. After around 3–6 months, the priorities change to preservation of transplant function and avoiding the long-term complications of immunosuppressive medication (the medication used to suppress the immune system to prevent rejection). The topics discussed include organization of outpatient follow up, immunosuppressive medication, treatment of acute and chronic rejection, and prevention of complications. The potential complications discussed include heart disease, infection, cancer, bone disease and blood disorders. There is also a section on contraception and reproductive issues.

Immediately after the introduction there is a statement of all the recommendations. These recommendations are written in a language that we think should be understandable by many patients, relatives, carers and other interested people. Consequently we have not reworded or restated them in this lay summary. They are graded 1 or 2 depending on the strength of the recommendation by the authors, and A-D depending on the quality of the evidence that the recommendation is based on.

## Abbreviations

ACEI: Angiotensin converting enzyme inhibitor
ACR: Albumin:creatinine ratio
ALERT: Assessment of LEscol in Renal Transplantation
AMR: Antibody mediated rejection
AMR: Antibody mediated rejection
APKD: Adult polycystic kidney disease
ARB: Angiotensin receptor blocker
ATG: Anti-thymocyte globulin
ATIIR: Angiotensin two receptor
AUC: Area under the curve
BCG: Bacillus Calmette–Guérin
BHSI: British Society for Histocompatibilty and Immunogentics
BKN: BK virus nephropathy
BMI: Body mass index
BPAR: Biopsy proven acute rejection
BTS: British Transplant Society
C_0_: Trough concentration
C_2_: Concentration at 2 h post dose
CAD: Coronary artery disease
CAMR: Chronic antibody mediated rejection
CIT: Cold ischaemic time
CKD: Chronic kidney disease
CKD-MBD: Chronic kidney disease mineral bone disorder
CMV: Cytomegalovirus
CNI(s): Calcineurin inhibitor(s)
CRF: Calculated reaction frequency
CVD: Cardiovascular disease
D-: Donor negative
D+: Donor positive
DCD: Donation after circulatory death
DEXA: Dual-energy X-ray absorptiometry
DM: Diabetes mellitus
DNA: Deoxyribonucleic acid
DNA: Did not attend
DoH: Department of Health
DSA: Donor specific antibody
EBV: Epstein Barr Virus
eGFR: Estimated glomerular filtration rate
ESRD: End stage renal disease
GRADE: Grades of Recommendation, Assessment, Development and Evaluation
GT: Glucose tolerance
HBsAb: Hepatitis B surface antibody
HCV: Hepatitis C virus
HDL: High-density lipoprotein
HEV: Hepatitis E virus
HIV: Human immunodeficiency virus
HSV: Herpes simplex virus
HT: Hypertension
IL2-RA: Interleukin-2 receptor antagonists
JBS: Joint British Societies
KDIGO: Kidney Disease Improving Global Outcomes
KTR(s): Kidney transplant recipient(s)
LDL: Low density lipoprotein
MI: Myocardial infarction
MMF: Mycophenolate mofetil
MPA: Mycophenolic acid
m-TORi(s): Mammalian target of rapamycin inhibitor(s)
NDSA: New donor specific antibody
NHSCSP: National Health Service Cancer Screening Programme
NICE: National Institute for Clinical Excellence
NMSC: Non-melanoma skin cancer
NSAIDs: Non-steroidal anti-inflammatory drugs
PCP: Pneumocystis pneumonia
PCR: Polymerase chain reaction
PCR: Protein:creatinine ratio
PML: Progressive multifocal leukoencephalopathy
PRA: Panel reactive antibody
PTDM: Post transplant diabetes mellitus
PTH: Parathyroid hormone
PTLD: Post transplant lymphoproliferative disorder
R-: Recipient negative
R+: Recipient positive
RANK: Receptor Activator of Nuclear Factor κ B
RAS: Renin angiotensin system
RCP: Royal College of Physicians
RCT: Randomised controlled trial
RNA: Ribonucleic acid
RRT: Renal replacement therapy
SCAR: Subclinical acute rejection
TDAs: T-cell (lymphocyte) Depleting Antibodies
VHL: Von Hippel–Lindau
VZV: Varicella zoster virus
